# Effect of Therapeutic Drug Monitoring on Adherence and Blood Pressure: A Multicenter Randomized Clinical Trial

**DOI:** 10.1093/ajh/hpae059

**Published:** 2024-05-07

**Authors:** Lene V Halvorsen, Camilla L Søraas, Anne Cecilie K Larstorp, Ulla Hjørnholm, Vibeke N Kjær, Knut Liestøl, Arleen Aune, Eirik Olsen, Karl Marius Brobak, Ola U Bergland, Stine Rognstad, Nikolai R Aarskog, Sondre Heimark, Fadl Elmula M Fadl Elmula, Eva Gerdts, Rune Mo, Marit D Solbu, Mimi S Opdal, Sverre E Kjeldsen, Morten Rostrup, Aud Høieggen

**Affiliations:** Department of Nephrology, Oslo University Hospital Ullevål, Oslo, Norway; Section for Cardiovascular and Renal Research, Oslo University Hospital Ullevål, Oslo, Norway; Institute of Clinical Medicine, University of Oslo, Oslo, Norway; Section for Cardiovascular and Renal Research, Oslo University Hospital Ullevål, Oslo, Norway; Section for Environmental and Occupational Medicine, Oslo University Hospital Ullevål, Oslo, Norway; Section for Cardiovascular and Renal Research, Oslo University Hospital Ullevål, Oslo, Norway; Institute of Clinical Medicine, University of Oslo, Oslo, Norway; Department of Medical Biochemistry, Oslo University Hospital Ullevål, Oslo, Norway; Section for Cardiovascular and Renal Research, Oslo University Hospital Ullevål, Oslo, Norway; Section for Cardiovascular and Renal Research, Oslo University Hospital Ullevål, Oslo, Norway; Department of Informatics, University of Oslo, Oslo, Norway; Department of Clinical Science, University of Bergen, Bergen, Norway; Department of Heart Disease, Haukeland University Hospital, Bergen, Norway; Norwegian University of Science and Technology, Trondheim, Norway; Department of Emergency Medicine, St. Olav’s University Hospital, Trondheim, Norway; Metabolic and Renal Research Group, UiT, The Arctic University of Norway, Tromsø, Norway; Section of Nephrology, University Hospital of North Norway, Tromsø, Norway; Section for Cardiovascular and Renal Research, Oslo University Hospital Ullevål, Oslo, Norway; Section for Cardiovascular and Renal Research, Oslo University Hospital Ullevål, Oslo, Norway; Institute of Clinical Medicine, University of Oslo, Oslo, Norway; Department of Pharmacology, Oslo University Hospital Ullevål, Oslo, Norway; Section for Cardiovascular and Renal Research, Oslo University Hospital Ullevål, Oslo, Norway; Institute of Clinical Medicine, University of Oslo, Oslo, Norway; Department of Acute Medicine, Oslo University Hospital Ullevål, Oslo, Norway; Department of Nephrology, Oslo University Hospital Ullevål, Oslo, Norway; Section for Cardiovascular and Renal Research, Oslo University Hospital Ullevål, Oslo, Norway; Institute of Clinical Medicine, University of Oslo, Oslo, Norway; Section for Cardiovascular and Renal Research, Oslo University Hospital Ullevål, Oslo, Norway; Department of Clinical Science, University of Bergen, Bergen, Norway; Department of Heart Disease, Haukeland University Hospital, Bergen, Norway; Department of Emergency Medicine, St. Olav’s University Hospital, Trondheim, Norway; Department of Cardiology, St. Olav's University Hospital, Trondheim, Norway; Metabolic and Renal Research Group, UiT, The Arctic University of Norway, Tromsø, Norway; Section of Nephrology, University Hospital of North Norway, Tromsø, Norway; Institute of Clinical Medicine, University of Oslo, Oslo, Norway; Department of Pharmacology, Oslo University Hospital Ullevål, Oslo, Norway; Department of Nephrology, Oslo University Hospital Ullevål, Oslo, Norway; Section for Cardiovascular and Renal Research, Oslo University Hospital Ullevål, Oslo, Norway; Institute of Clinical Medicine, University of Oslo, Oslo, Norway; Department of Cardiology, Oslo University Hospital Ullevål, Oslo, Norway; Section for Cardiovascular and Renal Research, Oslo University Hospital Ullevål, Oslo, Norway; Department of Acute Medicine, Oslo University Hospital Ullevål, Oslo, Norway; Department of Behavioral Sciences, Institute of Basic Medical Sciences, University of Oslo, Oslo, Norway; Department of Nephrology, Oslo University Hospital Ullevål, Oslo, Norway; Section for Cardiovascular and Renal Research, Oslo University Hospital Ullevål, Oslo, Norway; Institute of Clinical Medicine, University of Oslo, Oslo, Norway

**Keywords:** adherence, antihypertensive drugs, blood pressure, hypertension, nonadherence, randomized clinical trial, therapeutic drug monitoring

## Abstract

**BACKGROUND:**

Drug concentration in blood or urine is an acknowledged method to detect nonadherence. Observational studies suggest that informing patients about low or absent serum drug levels improves blood pressure (BP). We performed a multicenter randomized clinical trial to test the hypothesis that therapeutic drug monitoring (TDM) could improve drug adherence and BP in patients with uncontrolled hypertension (HT).

**METHODS:**

Patients were ≥18 years on stable treatment with at least 2 antihypertensive agents. We planned to randomize 80 nonadherent patients with a systolic daytime ambulatory BP ≥135 mm Hg to TDM intervention or not. The control group and the study personnel who measured BP remained uninformed about serum drug measurements throughout. All patients and physicians were blinded for BPs. Lifestyle advice and detailed information on the disease process and the importance of BP treatment were given to both groups.

**RESULTS:**

From 2017 to 2022, we randomized 46 diagnosed nonadherent from a total of 606 patients with uncontrolled HT. The TDM group had a 6.7 (±14.5) mm Hg reduction from 147.9 (±10.3) to 141.1 (±14.1) mm Hg, and the control group experienced a 7.3 (±13.2) mm Hg reduction from 147.1 (±9.2) to 139.1 (±17.4) mm Hg, *P* = 0.9 between groups. Adherence improved in both groups, 73% in the TDM group and 59% in the control group became adherent at 3 months, *P* = 0.51.

**CONCLUSIONS:**

In our prospective multicenter clinical trial of uncontrolled and nonadherent hypertensive patients, we found no additional effect of TDM on BP and drug adherence compared with standard care.

**CLINICAL TRIALS REGISTRATION:**

Trial Number NCT03209154, www.clinicaltrials.gov.

## Graphical Abstract

**Graphical Abstract:**
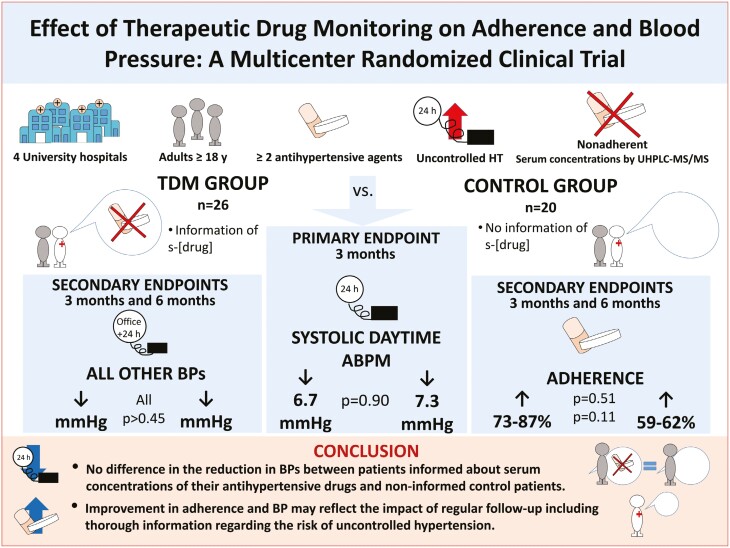

Nonadherence remains a major obstacle in hypertension (HT) treatment efficacy^1^ as well as in cardiovascular diseases in general^2^ reflected by recent guidelines.^3–6^ Measurement of drug concentration in urine or serum using ultra-high-performance liquid chromatography–tandem mass spectrometry (UHPLC–MS/MS) has become a reliable method to diagnose nonadherence.^7–10^ Whether information on analyses of antihypertensive drugs in blood or urine may improve adherence and subsequent blood pressure (BP) control is unknown.^11^ It has been suggested that informing nonadherent patients of their low or undetectable serum drug levels combined with counseling to overcome barriers to adherence may improve BP control.^12,13^ However, this has not been investigated in a prospective randomized controlled trial (RCT).

The concentration of drugs in blood is determined by dosage, absorption, distribution, metabolism, excretion, and drug interactions. Increasing the knowledge of pharmacokinetic and -dynamic properties of antihypertensive drugs may improve individualization of treatment and shared decision-making. Therapeutic concentration ranges for antihypertensive drugs are sparse, but we have previously defined trough-value dose-related serum reference ranges.^7,14^ The lower limit of these ranges can be used to define drug adherence.

We performed a nationwide multicenter, prospective RCT to test the hypothesis whether repeated standardized information on measurements of antihypertensive drugs in serum (TDM information) could improve drug adherence and subsequent BP control in proven nonadherent and uncontrolled hypertensive patients.

## METHODS

### Ethical considerations and data management

The study protocol was approved by the Regional Ethical Committee, conducted in accordance with the Helsinki declaration, and overseen by an independent Safety Monitoring Board. Participation was free of charge, and patients were not paid. All participants provided written informed consent which, as approved by the Ethical Committee, did not detail that drug concentrations would be measured in standard blood tests. See Supplementary Text §1 online for English extract of essential information in the patient information leaflet. Electronic data capture was managed with ViedocTM (PCG Solutions, Uppsala, Sweden), approved by the local Data Protection Officer.

### Data collection

**Figure 1.**
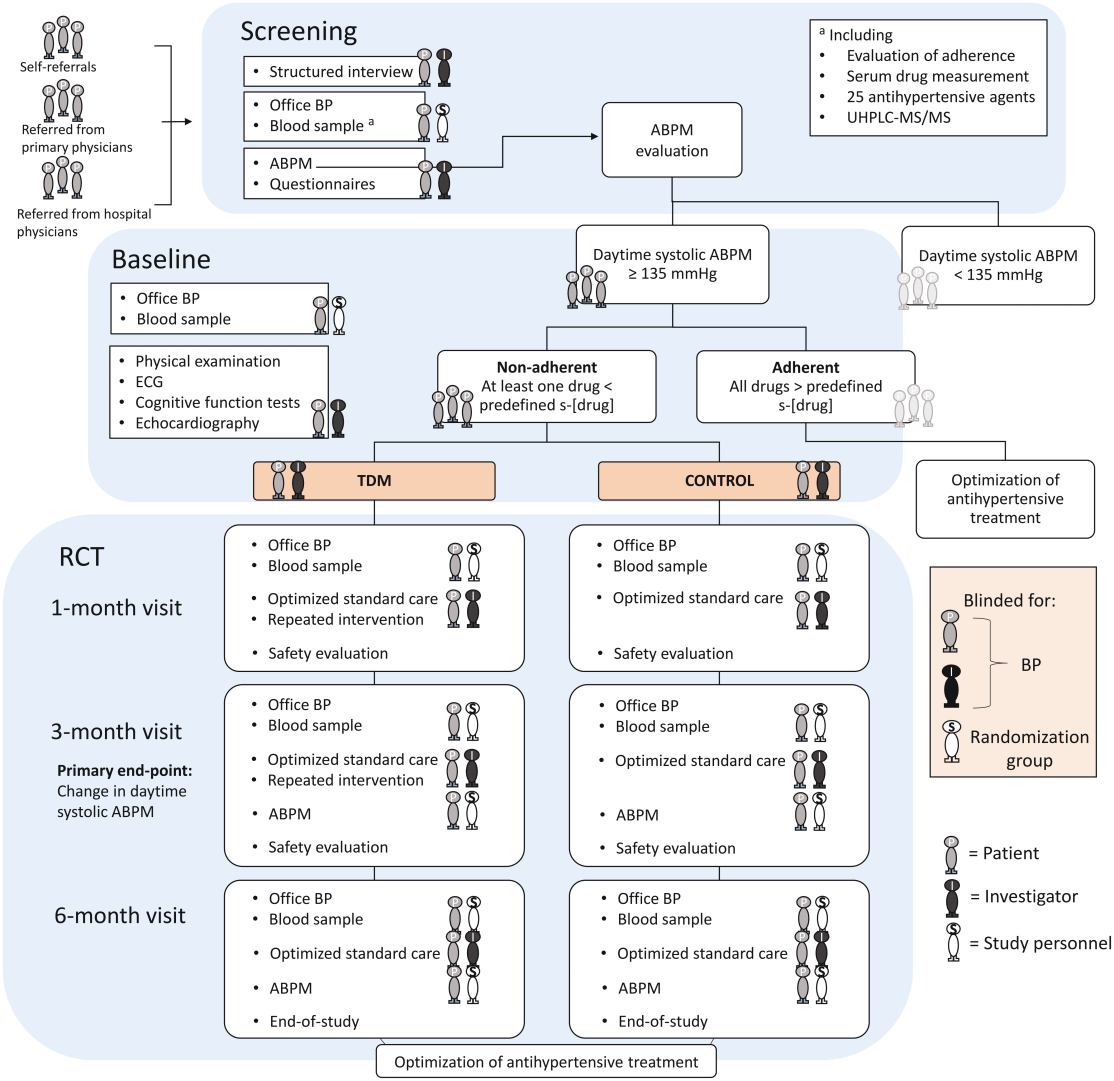
Study design. Abbreviations: ABPM, ambulatory blood pressure measurement; ECG, electrocardiogram; TDM, therapeutic drug monitoring; UHPLC–MS/MS, ultra-high-performance liquid chromatography–tandem mass spectrometry.

From 2017 to 2022, 1,156 hypertensive patients were investigated for eligibility to participate in our RCT (Figure 1). Patients were included at university hospitals across Norway, with 629 patients (54%) included at Oslo University Hospital, Ullevål, Oslo, 218 patients (19%) at Haukeland University Hospital, Bergen, 149 patients (13%) at the University Hospital of North Norway, Tromsø, and 160 (14%) at St. Olav’s University Hospital, Trondheim. Six hundred and sixty-two patients (57%) were referred from primary physicians and 176 patients (15%) from secondary or tertiary specialist centers. In addition, 313 patients (27%) contacted our research units directly, in response to advertisements in local newspapers, television, and social media. Patients did not receive any specific instructions regarding drug intake prior to the first study visit, in order to avoid influencing their drug adherence. A detailed description of study inclusion has previously been published.^7^ Of the 1,156 patients screened for eligibility, 606 (52%) with uncontrolled HT (including 192 with masked HT) were invited to a subsequent baseline visit.

According to the protocol inclusion ended in March 2022, after being extended 16 months due to the Covid pandemic. All 6-month visits were completed by August 2022 and queries were resolved by the end of January 2023.

### Inclusion and exclusion criteria

Inclusion was performed in a stepwise fashion: Patients were included if they were ≥18 years old, being prescribed ≥2 antihypertensive agents (or ≥1 fixed-dose combination pill), and with stable treatment for at least 4 weeks. See Supplementary Text §1 online for exclusion criteria. For the baseline visit, an additional inclusion criteria of systolic daytime ambulatory blood pressure (ABPM) ≥135 mm Hg was required, and those with systolic daytime ABPM ≥170 mm Hg or the disclosure of any exclusion criteria, were not invited to this visit. Nonadherent patients who fulfilled all criteria at the baseline visit were included in the RCT. Further details are provided in Supplementary Text §1 online.

### Screening visit

Participants were examined^7^ and told that they were taking part in a study aiming to improve BP control and that they might become eligible to participate in an RCT that required them to be blinded to their BP readings. They remained uninformed about the intention to screen for nonadherence with serum samples for drug analyses, as well as criteria for inclusion in the RCT, randomization, and intervention, and underwent a structured physician–patient interview including detailed information on current antihypertensive and concomitant drug treatment (Supplementary Text §2 online). Urine and blood samples were collected (Supplementary Text §3 online), thereafter we measured office BP and ABPM, and patients completed questionnaires.

### Baseline visit

Patients with uncontrolled HT (systolic daytime ABPM ≥135 mm Hg) were invited to the baseline visit, approximately 2 weeks after the screening visit. Based on the results of the serum concentration measurements, patients were defined as either adherent or nonadherent to prescribed antihypertensive drugs. The baseline visit included physical examination, electrocardiogram, echocardiography, evaluation of cognitive function, and aldosterone and renin analyses.

### Randomization

The nonadherent patients were randomized 1:1 in ViedocTM at the baseline visit to the intervention group; called the therapeutic drug monitoring (TDM) group, or the control group. Block randomization was organized by an independent statistician, with random block sizes and stratified by the study center.

### Primary endpoint and RCT visits

The primary endpoint was the difference in daytime systolic ABPM between groups at 3 months. Other BPs and adherence status were secondary endpoints.

Both groups attended the same number of visits during the 6-month follow-up (Figure 1) and antihypertensive medication regimens remained unchanged. Serum drug concentrations were measured at all follow-up visits. Office BP, sodium, potassium, and kidney function were measured for safety reasons in both groups at the 1-month visit in case nonadherent patients would start taking their renin–angiotensin–aldosterone system inhibitors or diuretics.

The 3- and 6-month visits included a full physical examination, electrocardiogram, ABPM, and routine blood and urine tests, and questionnaires were repeated. Echocardiography was repeated at the 6-month visit.

### TDM intervention

The patients in the TDM group were informed of the results of serum drug concentrations in a standardized non-prejudicing manner at the baseline visit, and this information was repeated at the 1- and 3-month visits. All investigators gave the TDM information according to preplanned detailed instructions in a standard operative procedure (Supplementary Text §4 online). The standard operative procedure opened up for individual advice based on the patients’ reactions and specific barriers to adherence, to ensure a good doctor–patient dialog. The investigator recorded the patients’ reactions to the TDM information immediately after the baseline visit. The control group remained uninformed that serum drug concentrations were measured and of the results of these analyses until after completion of the study.

### Optimized standard care

Both groups were by the same investigators, given identical lifestyle advice, information about the necessity of BP treatment, how to remember to take medications, and general information regarding complications, e.g., HT-mediated organ damage (Supplementary Text §4 online).

### BP measurements

Office BPs were measured by study personnel blinded for the randomization groups, with a validated device (Supplementary Text §5 online) in a standardized manner according to the European Hypertension guidelines.^15,16^ The same device was used for ABPM at the screening, 3-, and 6-month visits. The device was programmed to inflate every 20 minutes during the day, and every 30 minutes at night. For reading to be valid, at least 70% of measurements had to be successful, and no more than 2 subsequent hours without recordings could be present during the daytime.^7^ The investigators and patients were blinded to BPs throughout.

### Evaluation of adherence

The Department of Pharmacology, Oslo University Hospital, performed all pharmacological analyses. Measurements of serum drug concentrations were available for 25 of the most commonly prescribed antihypertensive agents in Norway using UHPLC–MS/MS (6490 Triple Quad LC/MS, Agilent Technologies Inc., Santa Clara, CA).^14,17^ An experienced clinical pharmacologist, masked for all variables except for current medication, gender, and age, classified the adherence status of the patient taking into consideration the dosage, reported time of last intake, and predefined serum reference ranges.^7,14^ Patients were defined as adherent if all prescribed antihypertensive agents were present in serum above the established cutoff value (the lower level of the reference range), and nonadherent if at least 1 agent was undetectable or below the cutoff (Supplementary Table S1 online).^7^ If in doubt, 2 clinical pharmacologists reviewed the results and reached a consensus on the adherence status. The consideration of adherence was not applied to loop diuretics (furosemide, bumetanide), as these drugs are undetectable in serum after 12–24 hours due to short half-lives.^18^

### Questionnaires

Patient-reported adherence to medications and patient-reported side effects were completed by the patients at the screening, 3-, and 6-month visits (Supplementary Text §6 online).

### Safety evaluation

A Safety Monitoring Board with 2 independent and experienced physicians was alerted via the data capturing system, Viedoc, and reviewed BPs above the safety threshold and severe adverse events (i.e., hospitalizations regardless of cause). If needed, study personnel contacted the Safety Monitoring Board directly regarding e.g., high BPs. Investigators monitored all patients closely with regard to clinical chemistry, new or worsening of side effects of antihypertensive drugs, and new symptoms and diagnoses during follow-up.

### Sample size determination

The sample size was estimated for the primary objective: to demonstrate a difference in daytime systolic ABPM between groups at 3 months. To detect a difference of ≥8 mm Hg, which we considered clinically relevant, we estimated that the study required 40 patients per group at an alpha-risk of 5% and a statistical power of 80% in a 2-sided *t*-test, assuming an SD of 13 mm Hg (Supplementary Text §7 online).

### Statistical analyses

We used IBM SPSS Statistics 26 (SPSS, Chicago, IL) for statistical analyses. Continuous variables are presented as mean (SD) or median (interquartile range), and categorical variables are presented as absolute numbers with percentages. We tested for normality using the Kolmogorov–Smirnov test. We tested for differences between groups using the Student’s *t*-test for normally distributed continuous variables or the Mann–Whitney *U*-test for those non-normally distributed. For categorical variables, we used the chi-square, Fisher’s exact, or the McNemar’s test. A 2-sided *P* < 0.05 was considered significant. Changes in continuous variables over time were tested by mixed analysis of variance.

## RESULTS

**Figure 2.**
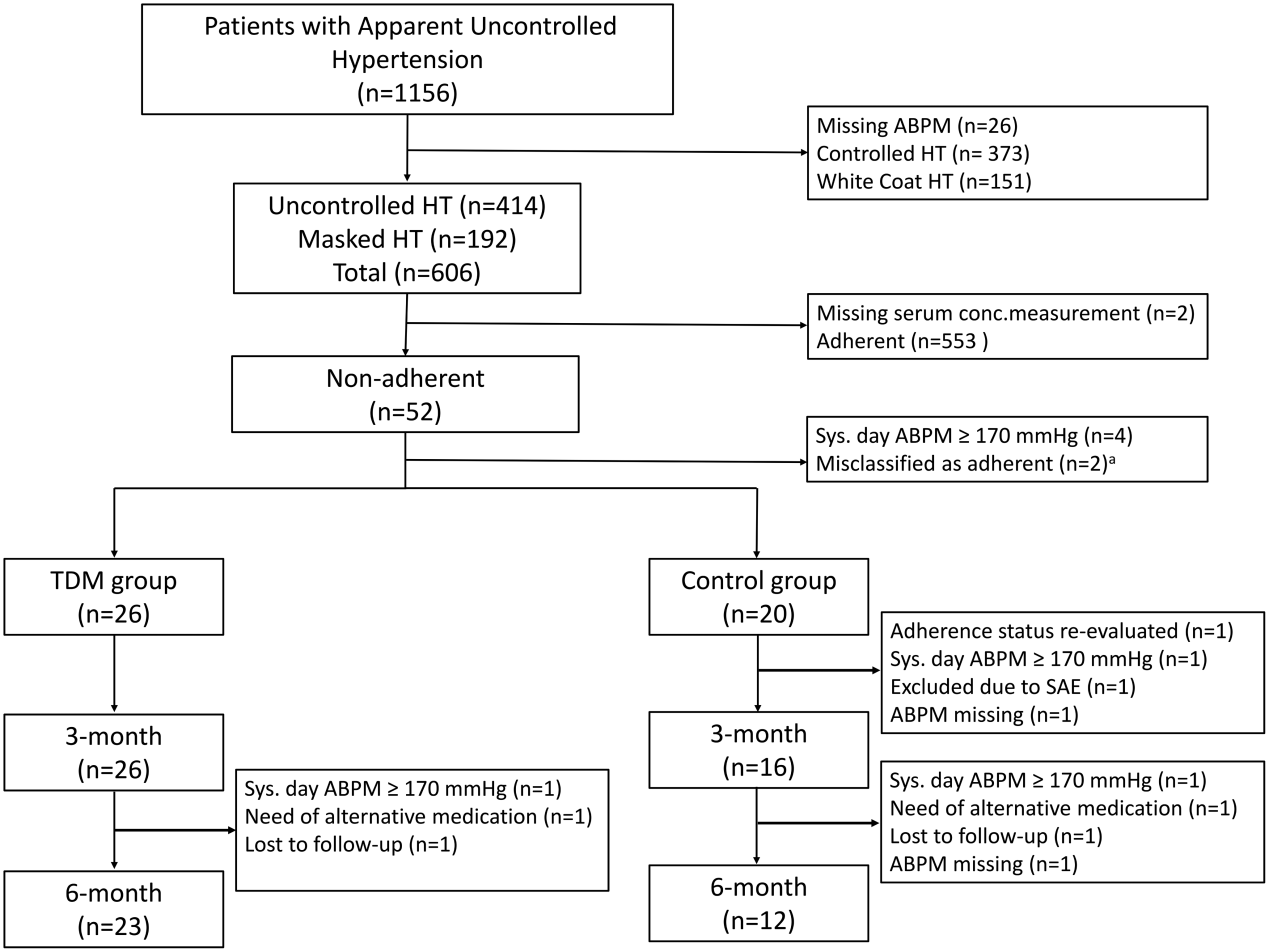
Patient flowchart. Abbreviations: ABPM, ambulatory blood pressure measurement; BP, blood pressure; RCT, randomized controlled trial; SAE, serious adverse event; TDM-info, standardized information on the patients’ serum concentrations, the intervention. ^a^These 2 patients had uncontrolled hypertension and were invited to the baseline visit, however, they were misclassified as adherent, thus not randomized and their antihypertensive medication was instead changed. A reevaluation by the pharmacologist disclosed that the serum drug concentrations did not match the evaluation and their adherence status was corrected to nonadherent, but they were no longer eligible for the RCT.

After a meticulous examination of 1,156 patients, we diagnosed 52 nonadherent patients with uncontrolled HT; 46 of these patients were randomized into the present RCT, and 6 patients were not randomized mainly due to BP above the safety threshold (daytime systolic ABPM ≥170 mm Hg) (Figure 2).

Twenty-six patients were randomized to TDM information, and 20 patients to the control group. The imbalance owes to block randomization. BPs, number of pills, and other characteristics were similar in the 2 randomized groups (Table 1). Three patients in the control group did not attend the 3-month visit: 1 was excluded at the 1-month visit due to BP above the safety threshold, 1 was hospitalized at baseline due to heart failure (serious adverse event), and 1 was not truly nonadherent since the pharmacy had changed the medication due to delivery failure. A fourth patient attended the 3-month visit but was not able to complete the ABPM and hence excluded (Figure 2).

**Table 1.** Baseline characteristics of the randomized patients

Variable	TDM group (*n* = 26)	Control group (*n* = 20)	*P* value
Females, *n* (%)	8 (31)	5 (25)	0.92
Age, y	57.1 (±11.1)	59.0 (±12.7)	0.59
BMI, kg/m^2^	30.4 (±5.4)	29.8 (±5.0)	0.73
Time since diagnosis of hypertension, y	12.9 (±8.8)	10.9 (±8.5)	0.43
Creatinine, µmol/l	75.0 (±16.4)	80.8 (±26.9)	0.37
eGFR, ml/min/1.73 m^2^	91.6 (±14.0)	82.8 (±21.4)	0.12
uACR, mg/mmol	5.2 (±14.9)	3.8 (±9.2)	0.72
uACR ≥3 mg/mmol, *n* (%)	6 (23)	4 (20)	>0.99
Left ventricular hypertrophy in ECG^a^, *n* (%)	7 (27)	4 (20)	0.73
Coronary artery disease, *n* (%)	4 (17)	3 (18)	>0.99
Atrial fibrillation, *n* (%)	2 (8)	2 (10)	>0.99
Diabetes mellitus type II, *n* (%)	3 (12)	3 (16)	0.69
Prescribed daily medications, mean number (SD)			
Antihypertensive pills	2.7 (±1.4)	2.7 (±1.6)	0.86
Antihypertensive agents	3.4 (±1.2)	3.1 (±0.9)	0.32
Concomitant agents	3.3 (±3.0)	3.2 (±2.4)	0.93
Total number of agents^b^	6.7 (±3.8)	6.3 (±2.9)	0.70
Total number of daily pills^c^	5.6 (±4.1)	5.1 (±2.8)	0.62
Patients prescribed antihypertensive combination pills, *n* (%)			
Only single-agent pills	8 (31)	8 (40)	0.73
≥1 fixed-dose combination pill	18 (69)	12 (60)	0.73
Only 1 antihypertensive pill	4 (15)	4 (20)	0.71
Nonadherence			
Completely nonadherent, *n* (%)	4 (15)	4 (20)	0.71
Partially nonadherent, *n* (%)	22 (85)	16 (80)	0.71
Self-reported nonadherence, score 5–25, median (IQR)	6 (5.7)	6 (5.7)	0.46

Results are reported as *n* (%), mean (±SD), or median (IQR), *P* value denotes differences between groups. Abbreviations: BMI, body mass index; ECG; electrocardiogram; eGFR, estimated glomerular filtration rate (CKD-EPI); IQR, interquartile range; TDM, therapeutic drug monitoring; uACR, urine albumin–creatinine ratio.

^a^Based on Cornell voltage criteria. Sokolow–Lyon criteria negative in all randomized patients.

^b^All antihypertensive agents + all concomitant agents.

^c^All antihypertensive pills + all concomitant pills.

### Primary endpoint and other BPs

**Figure 3.**
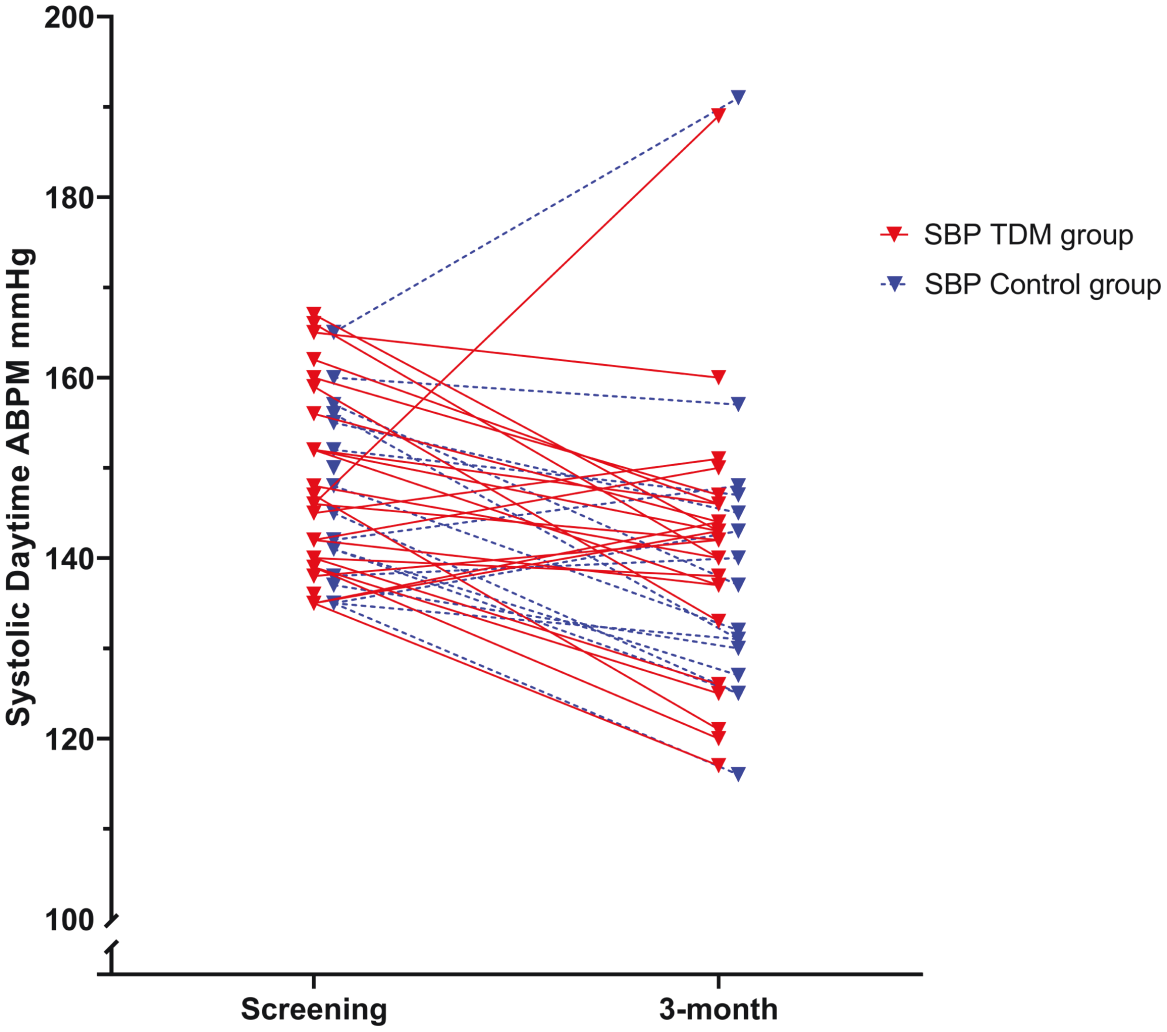
Individual patient data for the primary endpoint. Change in systolic daytime ABPM during 3 months in the intervention (solid line) and control (dotted line) groups. Visits are marked on the *X*-axis, and SBP on the *Y*-axis (mm Hg). The *Y*-axis is truncated. Abbreviations: ABPM, ambulatory blood pressure measurement; SBP, systolic blood pressure.

There was no difference in daytime systolic ABPM between the groups at 3 months (the primary endpoint). The TDM group had a 6.7 (±14.5) mm Hg reduction from 147.9 (±10.3) to 141.1 (±14.1) mm Hg, and the control group experienced a 7.3 (±13.2) mm Hg reduction from 147.1 (±9.2) to 139.1 (±17.4) mm Hg, *P* = 0.9 between groups (Figure 3 and Table 2). Daytime diastolic ABPM reduced from 89.5 (±10.2) to 86.7 (±10.9) mm Hg in the TDM group and from 86.7 (±9.8) to 82.8 (±13.1) mm Hg in the control group, *P* = 0.46. Nighttime ABPM in the TDM group reduced from 128.0 (±15.8)/74.5 (±10.2) mm Hg to 125.1 (±13.1)/72.4 (±8.5) mm Hg and in the control group from 131.9 (±13.8)/74.7 (±9.0) to 125.0 (±16.4)/70.9 (±9.4) mm Hg, *P* = 0.49/0.50. There was no difference between the groups in office systolic/diastolic BP at the 3-month visit (*P* = 0.99/0.80). The main reductions in office BP were seen from screening to baseline, i.e., before randomization, and were more prominent in the TDM group. In the TDM group, there was a 12.6 (±18.6)/7.0 (±7.8) mm Hg reduction from screening to baseline, and in the control group 5.7 (±13.4)/4.8 (±6.2) mm Hg, *P* = 0.17/0.3 (Supplementary Figure S1 online).

**Table 2.** Adherence and blood pressures at screening, 3 months, and the change from screening to 3 months

Variable	TDM group (*n* = 26)	Control group (*n* = 16)^a^	Numeric Δ between groups	*P* value
Adherent at screening, *n*	0	0	0	n.a.
Adherent at 3 months, *n* (%)	19 (73)	10 (59)	9	0.51
Ambulatory daytime SBP at screening, mm Hg	147.9 (±10.3)	147.1 (±9.2)	0.8	0.79
Ambulatory daytime SBP at 3 months, mm Hg	141.1 (±14.1)	139.1 (±17.4)	2.0	0.68
Δ from screening to 3 months, mm Hg	−6.7 (±14.5)	−7.3 (±13.2)	0.6	0.90
Ambulatory daytime DBP at screening, mm Hg	89.5 (±10.2)	86.7 (±9.8)	2.8	0.35
Ambulatory daytime DBP at 3 months, mm Hg	86.7 (±10.9)	82.8 (±13.1)	3.9	0.31
Δ from screening to 3 months, mm Hg	−2.9 (±8.9)	−4.9 (±7.7)	2.0	0.46
Ambulatory daytime HR at screening, bpm	76.7 (±12.6)	74.4 (±9.8)	2.3	0.50
Ambulatory daytime HR at 3 months, bpm	75.0 (±12.7)	70.2 (±9.7)	4.8	0.19
Δ from screening to 3 months, bpm	−1.7 (±6.5)	−3.0 (±5.1)	1.3	0.50
Ambulatory 24-h SBP at screening, mm Hg	142.7 (±10.1)	143.2 (±10.4)	0.5	0.88
Ambulatory 24-h SBP at 3 months, mm Hg	136.8 (±12.4)	135.8 (±16.2)	0.5	0.82
Δ from screening to 3 months, mm Hg	−6.0 (±14.9)	−6.3 (±13.1)	0.3	0.94
Ambulatory 24-h DBP at screening, mm Hg	85.5 (±9.3)	83.6 (±9.4)	1.9	0.48
Ambulatory 24-h DBP at 3 months, mm Hg	82.4 (±9.5)	79.9 (±11.0)	2.5	0.45
Δ from screening to 3 months, mm Hg	−3 (±8.7)	−4 (±7.3)	1.0	0.68
Ambulatory 24-h HR at screening, bpm	73.5 (±11.5)	72.4 (±9.1)	1.1	0.72
Ambulatory 24-h HR at 3 months, bpm	72.4 (±11.5)	69.1 (±8.7)	3.3	0.33
Δ from screening to 3 months, bpm	−1.1 (±5.4)	−1.6 (±5.7)	0.5	0.77
Ambulatory nighttime SBP at screening, mm Hg	128.0 (±15.8)	131.9 (±13.8)	3.9	0.39
Ambulatory nighttime SBP at 3 months, mm Hg	125.1 (±13.1)	125.0 (±16.4)	0.1	0.98
Δ from screening to 3 months, mm Hg	−2.9 (±18.9)	−6.8 (±14.7)	3.9	0.49
Ambulatory nighttime DBP at screening, mm Hg	74.5 (±10.2)	74.7 (±9.0)	0.2	0.95
Ambulatory nighttime DBP at 3 months, mm Hg	72.4 (±8.5)	70.9 (±9.4)	1.5	0.60
Δ from screening to 3 months, mm Hg	−2.1 (±10.3)	−4.2 (±8.6)	2.1	0.50
Ambulatory nighttime HR at screening, bpm	64.9 (±9.9)	67.4 (±10.9)	2.5	0.44
Ambulatory nighttime HR at 3 months, bpm	65.6 (±11.1)	64.2 (±7.8)	1.4	0.67
Δ from screening to 3 months, bpm	0.7 (±4.8)	−0.1 (±7.9)	0.8	0.75
Office SBP at screening, mm Hg	156.7 (±18.5)	154.3 (±17.0)	2.4	0.66
Office SBP at 3 months, mm Hg	143.4 (±16.1)	141.4 (±21.1)	2.0	0.73
Δ from screening to 3 months, mm Hg	−13.3 (±20.2)	−13.4 (±18.3)	0.1	0.99
Office DBP at screening, mm Hg	94.2 (±14.1)	94.3 (±12.0)	0.1	>0.99
Office DBP at 3 months, mm Hg	85.2 (±13.3)	84.7 (±13.1)	0.5	0.89
Δ from screening to 3 months, mm Hg	−9.0 (±14.2)	−10.0 (±10.0)	1.0	0.80
Office HR at screening, bpm	70.8 (±13.8)	71.9 (±10.1)	1.1	0.77
Office HR at 3 months, bpm	72.7 (±12.7)	71.7 (±12.0)	1.0	0.78
Δ from screening to 3 months, bpm	1.9 (±7.8)	1.1 (±6.9)	0.8	0.71
Ambulatory daytime SBP <135 mm Hg at screening, mm Hg	0	0	0	*n*.a.
Ambulatory daytime SBP <135 mm Hg at 3 months, *n* (%)	6 (23)	8 (50)	2	0.14

The primary endpoint was systolic daytime ABPM. Results are reported as *n* (%) or mean (±SD), *P* value denotes the difference between the groups. Abbreviations: ABPM, ambulatory blood pressure measurement; BPM, beats per minute; DBP, diastolic blood pressure; HR, heart rate; SBP, systolic blood pressure; TDM, therapeutic drug monitoring; Δ, change; n.a., not applicable.

^a^Office BP and adherence evaluation performed in 17 patients, ABPM missing for 1 patient in the control group at the 3-month visit.

There was no difference in BP reduction between the groups (Supplementary Figure S2 online and Table 2), not even when including patients excluded or lost to follow-up before the 3-month visit (intention-to-treat), using their last measured BP in the statistical analyses (Supplementary Table S2 online).

**Figure 4.**
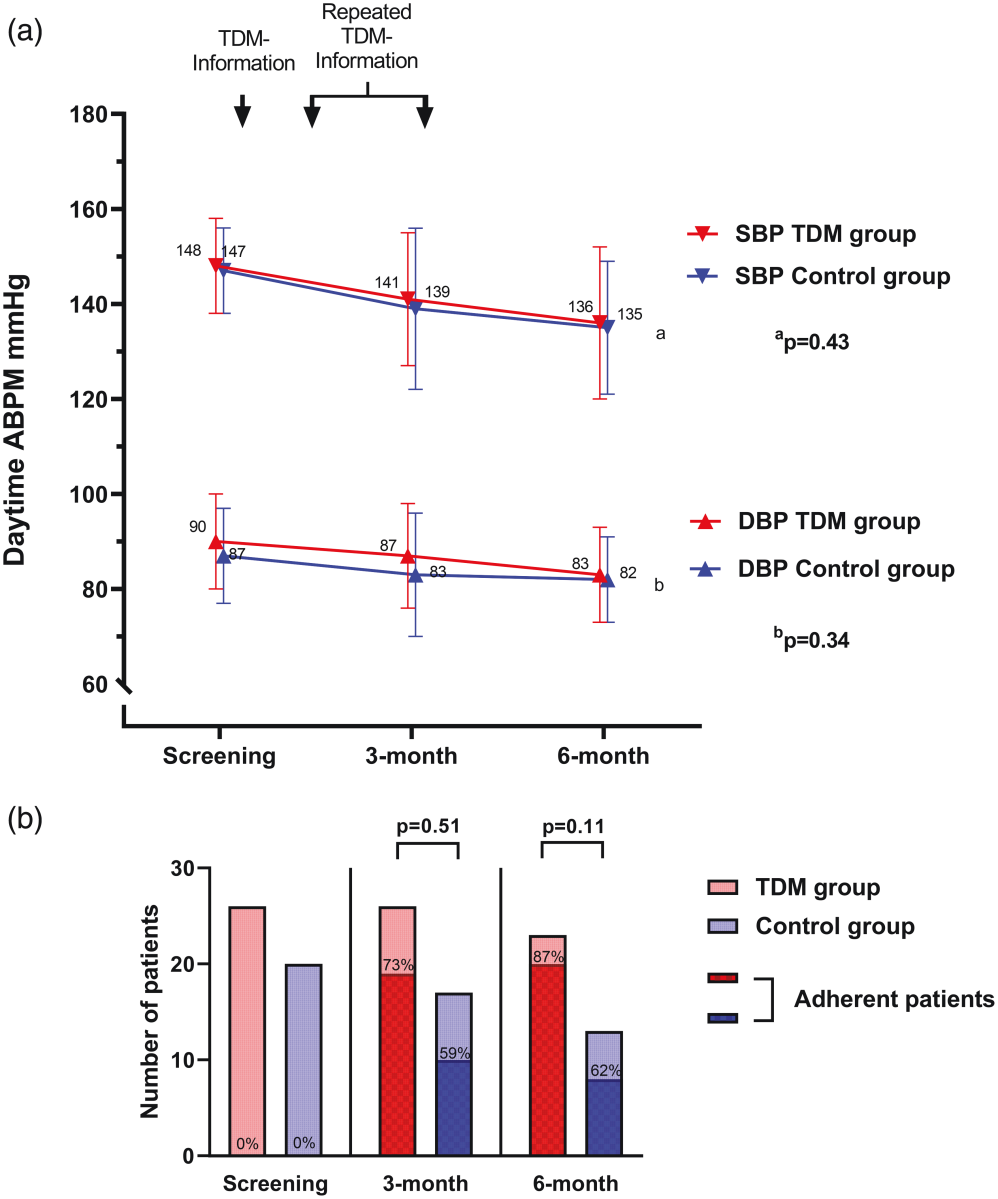
Daytime ABPM and adherence during 6-month follow-up. Panel **a**: Mean blood pressure (mm Hg) is shown on the *Y*-axis (truncated). TDM group in red and the control group in blue. Visits are marked on the *X*-axis. Daytime systolic ABPM (*P* = 0.43)^a^ and diastolic ABPM (*P* = 0.34)^b^ did not differ between the groups over time (ANOVA). Panel **b**: The number of patients is shown on the *Y*-axis, those attending the visit in red (TDM group) or blue (control group), and the number of adherent patients in the squared pattern. Differences between groups are not significant with Fisher’s exact test. Abbreviations: ABPM, ambulatory blood pressure measurement; ANOVA, analysis of variance; DBP, diastolic blood pressure; SBP, systolic blood pressure; TDM-info, standardized information on the patients’ serum concentrations.

There was a nonsignificant further drop in BP from 3 to 6 months, but still no difference between the groups; daytime systolic ABPM in the TDM group being 136 (±16) mm Hg, and in the control group 135 (±14) mm Hg, *P* = 0.79 (Figure 4 and Supplementary Table S3 online ). Neither analysis including the 11 patients not attending the 6-month visit shows any difference in BPs (Supplementary Table S4 online).

### Adherence

Adherence to antihypertensive medication improved in both groups. More patients in the TDM group (73%) vs. the control group (59%) became adherent at 3 months, although not statistically significant (*P* = 0.51). At the 6-month visit, corresponding percentages were 87% vs. 62% (*P* = 0.11) (Figure 4 and Supplementary Figure S3 online). All patients but one in the TDM group self-reported good adherence, and there was no significant difference between groups at inclusion, *P* = 0.46 (Table 1) or throughout (Supplementary Text §8 online).

At screening 8 patients (17%) were completely nonadherent, i.e., none of their prescribed antihypertensive medications were present in blood samples. The rest of the patients were partially nonadherent, i.e., nonadherent for 1 or more of their antihypertensive drug(s), but not for all. After 3 months only 2 patients were completely nonadherent, both in the TDM group, and after 6 months, 3 patients were completely nonadherent, 2 in the TDM group (not the same individuals as at the 3-month visit) and 1 in the control group.

### Side effects and safety evaluation

On direct questioning at the screening visit, 16 patients in each group reported side effects that they related to their antihypertensive medication (62% vs. 80%, *P* = 0.21). There were no differences in the number or severity of side effects or changes during follow-up (Supplementary Table S5 online). Three patients were excluded from the study due to high BP, and 2 patients were excluded due to a need for other medication adjustments (Figure 2). One patient was excluded due to heart failure (severe adverse event), the Safety Monitoring Board concluded that this was related to many years of uncontrolled HT and unrelated to participation in the present study.

## DISCUSSION

We have performed an RCT to study the effect of TDM in nonadherent patients with uncontrolled HT in a double-blinded approach. Enrollment goals according to power calculations were not met, as we were able to include 46 out of 80 patients. Nevertheless, we found no difference in BP response compared with optimized standard care. However, drug adherence improved in both the TDM and the control group and BP decreased significantly, but similarly in both groups. These findings are in contrast to positive results previously described in observational studies.^12,13^

The number of randomized patients is a limitation as we did not reach the number of assessed patients in accordance with our power calculations, and therefore precludes definitive, generalizable statements. Ideally, this could be addressed in a larger follow-up multinational study, which would enhance recruitment and strengthen the generalizability of the findings.

We had to examine 1,156 patients thoroughly in order to randomize 46 patients. This reflects the challenge to recruit nonadherent patients voluntarily to a clinical study, which we believe is due to numerous reasons: (i) a strict definition of nonadherence, (ii) the possibility of white-coat adherence, (iii) potential resistance to attend studies for some nonadherent patients since there is a risk of being revealed, and (iv) the Covid-19 pandemic, especially during 2020 and 2021 (i.e., hospital entry rules during lock-down and patients not willing to travel to the hospital due to fear of SARS-CoV-2).

Our data clearly show that uncontrolled HT is common (i.e., 52%) in this Norwegian population of patients using at least 2 antihypertensive agents. However, the prevalence of nonadherence explaining uncontrolled HT is moderate, only 8.6% of *n* = 606 compared with the 29.3% which we previously detected in a study in apparent treatment-resistant HT comparing renal denervation with optimized medication.^19^ A study from a University Hospital in Paris^20^ included all patients who attended the HT outpatient clinic within 4 months. All patients gave informed consent prior to the appointment that explicitly stated that adherence measurement was performed in urine specimens. They found 15 nonadherent (12 partially nonadherent, 3 fully nonadherent) out of 174 participants, i.e., 8.6% nonadherent, a prevalence consistent with our current findings. Compared with studies with a higher prevalence of nonadherence,^19,21,22^ we could not offer a nonpharmacological intervention such as renal denervation to encourage patients to attend.^23,24^ Poor motivation for nonadherent patients to travel to and participate in this unpaid study involving several visits and potentially uncomfortable ABPM recordings, and an unknown intervention, may also possibly explain difficulties in recruitment. Patients who attend clinic visits regularly may not reflect the adherence patterns of patients who miss appointments. If we for future studies, could increase the percentage of patients with uncontrolled HT who miss scheduled follow-up appointments and measure drug levels in these individuals, it might be instructive regarding nonadherence in a more representative sample.

The finding of 90% adherence among uncontrolled hypertensive patients may also reflect high confidence or trust in physicians who have prescribed their drugs in both Norway and France. Most importantly this was explained by straightforward under-dosing of standard antihypertensive medication. Patients had known HT for many years, they had regular visits to their general practitioners with BP measurements, and renewal of prescriptions with all expenses covered by social healthcare, but still their BP was not controlled. Documented Inertia appeared in several major RCTs with altogether about 55,000 patients and interestingly, it appears that physician inertia is a more common cause of uncontrolled HT in our study population than nonadherence to the prescribed therapy.^25^

Objective methods of detecting nonadherence are needed, since subjective, self-reported nonadherence methods (questionnaires) do not correspond to the findings of serum concentration measurements, as we published.^7^ For considerations regarding our pharmacological method see Supplementary Text §9 online. With HT being the most important risk factor for cardiovascular disease and death in the world we would not hesitate to say that it is worthwhile measuring serum drug concentrations to detect the 8.6% who were diagnosed in this way. Furthermore, verifying true uncontrolled HT with the need for up-titration of the medication is an important finding in our study.

Detailed analyses^7^ could not identify typical predictor(s) of patients who were truly uncontrolled and nonadherent with 1 important exception; namely not using a single pill combination, by some of us shown to be a key variable.^26^ The wide inclusion criteria in the present study may have contributed to reduce differences between the adherent and nonadherent patients since the population is quite heterogeneous. Previous studies have shown that nonadherence is associated with an increased number of daily prescribed pills.^7,26^ We included patients prescribed ≥2 antihypertensive agents or ≥1 fixed-dose combination pill. Sixty-five percent of included patients used ≥1 combination pill, significantly more common in adherent patients (67%) vs. the nonadherent patients (45%), and the total number of prescribed antihypertensive pills was higher in the nonadherent patients.^7^ The high percentage of combination pills, further decreasing the number of tablets per day, maybe one of the reasons for the low prevalence of nonadherence in this study. Patients in our study were on average prescribed 3.3 antihypertensive agents per day, but not necessarily in maximum doses. Their medications were unchanged throughout the study period. Few of our nonadherent patients were completely nonadherent, most of them were only partially nonadherent. Low doses and simple regimens may not entail a significant BP reduction compared with if we only had included patients with apparent treatment-resistant HT or if we had optimized the patients’ medication regimens before inclusion. Yet, the study design with wide inclusion criteria implies that our results are generalizable for a broader hypertensive population which is frequently encountered both in primary care and hospital settings.

Fear of side effects is an important barrier to nonadherence that needs to be discussed between patient and physician. In clinical practice, TDM may offer an opportunity to explore this. In the setting of this clinical study, careful registrations of possible side effects were performed at every visit, and significant changes in severity or new symptoms warranted an evaluation by the study safety monitoring board. If patients were reporting severe side effects or fear of these so that changes in antihypertensive medication were indicated, they were not included or were taken out of the study. Keeping the antihypertensive regimens unchanged made improvement in BP possible to relate to better adherence.

However, 3 and even 6 months, maybe too short time to change patients’ opinions and behavior. Perhaps repeated serum concentrations, serum concentrations in combination with directly observed therapy and ABPM and dialog regarding these measurements may show the patients that adherence, serum concentrations, and BPs are related and hence gradually improve BP over time.

The intervention was standardized and aimed to explain in detail the need for medication adherence to increase serum concentrations and treatment effects. Adherence did improve and some of the patients in the TDM group started to take their medication after learning about the beneficial actions of the drugs, and that the drugs were absent in their blood. Since adherence improved in the control group to a similar extent, we assume that the improvement was mainly due to the general information given to them. Thus, our data suggest no additional effect of repeated TDM beyond that of optimized standard care given at frequent visits, for BP control. There is, however, a numeric difference in adherence between the groups, and the nonsignificance in adherence may be due to a lack of statistical power. The total improvement in adherence and BP throughout the study may reflect the importance of regular visits, thorough repeated information, and a beneficial patient–physician relationship in HT treatment. Thus, our findings indicate that these simple and low-cost factors may be as efficient as the more costly TDM analyses for a general hypertensive population. The optimized standard care given to both groups might be seen as an intervention by itself, however, consists solely of information supposed to already have been given to the patients as they already were treated for their HT. A proper control group and the double-blinded approach of our study could to a large extent explain the outcomes arguing against TDM being an effective intervention by itself. Previous observational studies^12,13^ describe an extensive BP reduction achieved by discussing the nonadherent results with all patients. By optimizing the general information given to the patients, we could test whether it was the TDM information itself or other aspects of the care that improved the BP, hence we find this comparison of importance. The fact that simply thorough information is enough to reduce BP, is important in areas with limited availability of concentration measurements.

We measured drug concentrations in blood in our study, while others may prefer urinalysis.^10^ An advantage of using serum instead of urine is that serum quantification may be used to assess drug response and guide drug treatment. Analyses of drugs in urine may detect the presence or absence of medication (qualitative assessment) but cannot be used to quantify improvements in adherence and may, therefore, not be as useful in the follow-up of the individual patient.

Furthermore, there is not even a small trend for the TDM concept to influence BP in as much as the changes in BP were literally identical between the TDM group and the non-informed control group. Improvements in BP (and adherence) were thus unspecific and likely caused by the broad information to *both* study groups on the importance of treatment of HT including a healthy lifestyle and taking medication precisely as prescribed to avoid complications. By “unspecific” we also include Hawthorne^27^ and time effects and other patients’ and investigators’ unspecific effects. Thus, we did an extensive attempt to neutralize this effect—including strict inclusion criteria of 4 weeks of stable medication before work-up and possible enrollment.

We strongly believe that the strengths of our study lie in strict standardization and thoroughness as described in the method section, as well as the multicenter- and double-blinded approach. As an example, the oral TDM information was given according to a written standard operative procedure (Supplementary Text §4 online) which only allowed minor improvisations depending on the patient’s response.

Even though we did not find evidence that TDM *per se* reduced BP, this objective and quantitative method to reveal nonadherence may open up the conversation between the patient and healthcare personnel, explain why BP goals are not reached, and improve adherence over time if the patient is motivated to overcome this very important barrier to BP control. All reasons for nonadherence may not have been disclosed in the setting of this study, and more knowledge is needed to make the best use of TDM information.

### Study limitations

Besides the already discussed limitation of not meeting the recruitment target, the fact that the investigators were not blinded to the serum concentrations in the control group might be considered a limitation. By the design of the study nonadherence at inclusion was known to the investigators at the time of randomization, and the same investigators gave the TDM information to the TDM group and the optimized standard care to both groups. Results of serum concentrations at follow-up visits were received 1–2 weeks after the visit and were thereby unlikely to affect the optimized standard care, as both the investigator and the patient were blinded for the primary endpoint namely BP, while the study personnel measuring BP were blinded for the randomization groups.

## Conclusions

In our prospective multicenter clinical trial of uncontrolled and nonadherent hypertensive patients, we found no additional effect of TDM compared with standard care. Our data, although we did not meet the enrollment goal of 80 patients, do not support the use of TDM in the follow-up to improve adherence and BP of nonadherent and uncontrolled hypertensive patients prescribed ≥2 antihypertensive agents. The improvement in BP control and adherence seen in both groups may be due to the optimized standard care given to all patients and may reflect the importance of regular visits, thorough repeated information, and a beneficial patient–physician relationship in HT treatment.

There is a need for further research on how to use TDM in HT care, and to identify patients where it might be clinically useful. In the future, TDM may be used to individualize antihypertensive treatment.

## SUPPLEMENTARY DATA

Supplementary materials are available at *American Journal of Hypertension* (http://ajh.oxfordjournals.org).

hpae059_suppl_Supplementary_Material

## Data Availability

The data that support the findings of this study are available from the corresponding author upon reasonable request.

## Notes

All authors take responsibility for all aspects of the reliability and freedom from bias of the data presented and their discussed interpretation.
