# Supplementary material for: Effect of Therapeutic Drug Monitoring on Adherence and Blood Pressure: A Multicenter Randomized Clinical Trial
Source: Am J Hypertens. 2024 May 7;37(10):826–36. doi: 10.1093/ajh/hpae059 (PMC11403020; doi:10.1093/ajh/hpae059)
Supplement: hpae059_suppl_Supplementary_Material [file hpae059_suppl_supplementary_material.docx]

**American Journal of Hypertension Ms. AJH-D-24-00064r.**

**ONLINE SUPPLEMENT**

**Effect of Drug Monitoring on Adherence and Blood Pressure: A Multicenter Randomized Clinical Trial**

Lene V. Halvorsen, MD^1,2,3,ID^, Camilla L. Søraas, MD, PhD^2,4,ID^, Anne Cecilie K. Larstorp, MD, PhD^2,3,5,ID^, Ulla Hjørnholm, MSc^2^, Vibeke N. Kjær^2^, Knut Liestøl, PhD^6,ID^, Arleen Aune, MD^7,8,ID^, Eirik Olsen, MD^9,10,ID^, Karl Marius Brobak, MD^11,12,ID^, Ola U. Bergland, MD, PhD^2^, Stine Rognstad, MD^2,3,13,ID^, Nikolai R. Aarskog, MD^2,3,14^, Sondre Heimark, MD^1,2,3,ID^, Fadl Elmula M. Fadl Elmula, MD, PhD^2,ID^, Eva Gerdts, MD, PhD^7,8,ID^, Rune Mo, MD, PhD^10,15,ID^, Marit D. Solbu, MD, PhD^11,12,ID^, Mimi S. Opdal, MD, PhD^3,13,ID^, Sverre E. Kjeldsen, MD, PhD^1,2,3,16,ID*^, Morten Rostrup, MD, PhD^2,14,17,ID^, Aud Høieggen, MD, PhD^1,2,3,ID^

**Affiliations:**
^1^Department of Nephrology, Oslo University Hospital Ullevål, Oslo, Norway; ^2^Section for Cardiovascular and Renal Research, Oslo University Hospital Ullevål, Oslo, Norway; ^3^Institute of Clinical Medicine, University of Oslo, Oslo, Norway; ^4^Section for Environmental and Occupational Medicine, Oslo University Hospital Ullevål, Oslo, Norway; ^5^Department of Medical Biochemistry, Oslo University Hospital Ullevål, Oslo, Norway; ^6^Department of Informatics, University of Oslo, Oslo, Norway; ^7^Department of Clinical Science, University of Bergen, Bergen, Norway; ^8^Department of Heart Disease, Haukeland University Hospital, Bergen, Norway; ^9^Norwegian University of Science and Technology, Trondheim, Norway; ^10^Department of Emergency Medicine, St.Olav`s University Hospital; Trondheim, Norwa ^11^Metabolic and Renal Research Group, UiT The Arctic University of Norway, Tromsø, NorwaY; ^12^Section of Nephrology, University Hospital of North Norway, Tromsø, Norway; ^13^Department of Pharmacology, Oslo University Hospital Ullevål, Oslo, Norway; ^14^Department of Acute Medicine, Oslo University Hospital Ullevål, Oslo, Norway; ^15^Department of Cardiology, Trondheim University Hospital, Norway; ^16^Department of Cardiology, Oslo University Hospital Ullevål, Oslo, Norway; ^17^Department of Behavioral Sciences, Institute of Basic Medical Sciences, University of Oslo, Oslo, Norway.

**Supplemental Methods**

**Online Suppl. Text §1**

**The essential key sentences of the language used in the patient information leaflet:**

-This is a request for you who are being treated for high blood pressure to participate in a study investigating how to improve the follow-up of patients with high blood pressure. International guidelines for blood pressure treatment state that patients and healthcare personnel should work together to achieve blood pressure treatment goals. It is possible to evaluate whether a patient receives the right treatment by measuring blood pressure, taking blood and urine samples, and registering eventual side effects of the medications.

- Study participants with high blood pressure who fulfill the study’s criteria for participation, are randomly assigned in two groups, which differ due to different follow-up.

- As long as you are a participant in the study no changes will be made in your medication. Changes are made only if medically required. In clinical practice changes in medication usually are made more quickly. It is known that high blood pressure can have many different causes, and in this study other factors than blood pressure per se are therefore also considered, and time is spent together with the participants to assess the different factors. Participation in the study differs from routine clinical care by invitation to extra visits, as well as extra examinations.

**Exclusion criteria**

Exclusion criteria were estimated glomerular filtration rate (eGFR) <30 mL/min/1.73m^2^, urine albumin/creatinine ratio (uACR)>300 mg/mmol, poor Norwegian language skills, pregnancy, admitted illicit drug abuse, or psychiatric disorders and impaired cognitive function that could limit the ability to evaluate the efficacy or safety of the protocol.

**Second screening visit**

If a patient at the screening visit was non-adherent and had a BP above our safety threshold (systolic daytime ABPM ≥170 mmHg), we adjusted their antihypertensive treatment and invited them to a second screening at least four weeks later, given that other inclusion criteria were met.

**Patients not included in the RCT**

For adherent patients participation in the study ended after the screening visit if their blood pressures were controlled, or after the baseline visit if their blood pressures were uncontrolled. Antihypertensive treatment was optimized before patients returned to their regular follow-up at their primary care physician or in the specialist care. Adherence status and ABPM were not repeated at the baseline visit and therefore we don’t have any further information on their adherence status or successive blood pressures.

**Online Suppl. Text §2**

**Number of antihypertensive agents and tablets**

We recorded each separate antihypertensive agent, along with the total number of prescribed antihypertensive pills per day – both single-agent pills and fixed-dose combination pills. We also recorded the patient-reported time of intake of last doses of antihypertensive medications and duration of treatment. The total number of prescribed daily antihypertensive and concomitant treatment was calculated by summation of all prescribed pills per week divided by seven days, in order to account for concomitant medication taken every other day or once a week. Antihypertensive medications prescribed in doses not possible to achieve with one tablet, counted as two (or three) tablets. In addition, we counted each separate concomitant agent to reflect also non-oral medications (such as inhaled, ophthalmic and transdermal medications etc.).

**Online Suppl. Text §3**

**Urine Sample and Blood Tests**

Patients delivered morning urine samples for analysis of uACR and biobanking. Following office BP measurement and before 24-hour ABPM, blood samples for hematological- and biochemical analyses were collected, as well as a 5 mL Vacutainer tube TM (BD, Franklin Lakes, NJ, USA) without additives for analyses of antihypertensive agents. If patients reported to use nifedipine, we covered the tubes with aluminum foil to prevent photodegradation. We centrifuged the tubes for 10 min at 3000 rpm, and pipetted serum into Sarstedt tubes (Sarstedt, Nümbrecht, Germany) before storing in the freezer at -20°C.

Estimated glomerular filtration rate was calculated by The Chronic Kidney Disease Epidemiology Collaboration (CKD-EPI) equation.

**Online Suppl. Text §4**

**Copy of instructions for the intervention**

**Title: Standardized TDM-information**

**Aim:** Explain how to convey information regarding results of serum concentration measurements to the patient. This is **THE INTERVENTION in the IDA Study.** It is of **high importance** to standardize the information, so that the intervention will be the same for all patients.

If the patient should receive the intervention – follow instructions on p. 1-3.

If the patient is in the control group – follow instructions on p. 4.

**Information to the intervention group**

Print the figure of serum concentration measurement before the visit. This is shown to the patient:


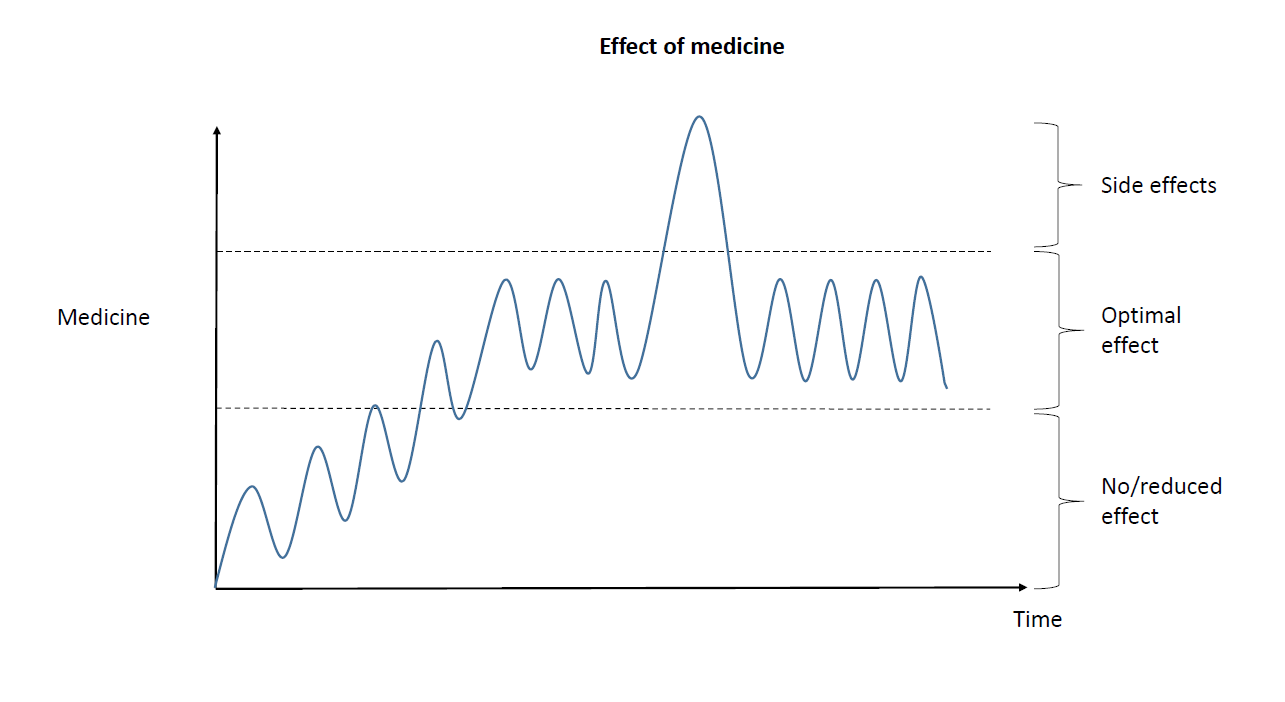


*The doctor explains simultaneously as pointing at the figure:*

«For antihypertensive drugs (or other drugs) to have effect, they have to be absorbed in the intestines and distributed throughout the body by the circulation. When a drug is absorbed, levels rise in the blood, and then decrease again towards the next dose. If you take your medication at the same time every day, the fluctuations decrease, and blood levels stabilize. The drug works/has effect in this level (the doctor points to «optimal effect» in the figure), at lower levels the effect is poorer, *(the doctor points to suboptimal effect)*, and high levels lead to more side-effects *(the doctor points to the upper part of the graph with side-effects).*

There is now a method that can measure the level of antihypertensive drugs in the blood, and the last time you were here we collected blood samples from you, and measured this. We found very little/no medication in your blood. The most common reason for this is that the medications are not used every day. “What are your thoughts about this?

In your opinion, how is this related to your blood pressure?”

No matter what the patient answers – return to the figure – repeat the explanation with their specific values, which are low. «Here is what we have measured. This is why your blood pressure remains elevated. Therefore it is important that you take your medication every day from now on.»

(If the patient asks directly – we confirm that we will take new blood samples during the study).

*Tips to further dialogue:*

*Give concrete individual advice on how to improve adherence depending on what is revealed in the conversation. Examples of how the patient may react:*

1. PROTEST: «I`ve been taking my medications every day (and you must have made a mistake).»
 *Answer: Open up to the possibilities of sources of error. Confirm that we also examine the patient for this.*

2. CONSENT: «I haven`t taken my medications…» Do not open for the possibility of CYP-polymorphisms.
 a) «...because I always forget to take them». Missed doses: Give advice on….  *Answer: Acknowledge that this can explain the low levels.*

b) «…because I don`t think they work/have effect»
 *Answer: «That (can) explain why the results are in this way. It is still important that you take your medication as agreed with your physician.»*

c) «…because I have side-effects»
  *Answer: Ask about the seriousness of the side-effects. If the patient doesn`t want to try to take the medication at all, he/she will have to be excluded and referred (back) to follow-up.*

d) «…because I don`t like to take medicines»
 *Answer: Acknowledge that that is understandable.*

3. EITHER NOR: «I sometimes forget to take them»
 *Answer: «That can explain the results of the blood sample.»*

(The doctor fills out patient reaction to TDM immediately after the visit in the appropriate form - see SOP 24.)

When the patient does not have more questions, the doctor continues with information regarding serious consequences:

**Serious consequences**
«You have probably been told earlier why it is crucial to take your medication, but it is important for me that you also hear it from me, so that I know what you have been told: High blood pressure is something you don`t notice you have. If you leave it untreated/undertreated for a long time, it can damage your internal organs, which can lead to serious consequences; examples are heart attack, heart failure, kidney failure and stroke. These diseases may affect your quality of life, and in worst case you may die from it.»

Finally the doctor gives general advice:

**Advice, tips and prevention**
«Do you know how to take your medication? /Do you know how to use your drugs?

In addition to taking you medication, there are several measures you can do to reduce your blood pressure; including a healthy diet and exercise. Particularly a high intake of salt may worsen the blood pressure, and even lead to a suboptimal effect of some of your medications (diuretics). Do you have any questions regarding this?»

Finally, the doctor gives general advice:

**Advice, tips and prevention**
«Do you know how to take your medication?

In addition to taking your medication, there are several measures you can do to reduce your blood pressure; including a healthy diet and exercises. Particularly a high intake of salt may worsen the blood pressure, and even lead to a suboptimal effect of some of your medications (diuretics). Do you have any questions regarding this?»

**Information to the control group**

**Serious consequences**
«You have probably been told earlier why it is crucial to take your medication, but it is important for me that you also hear it from me, so that I know what you have been told: High blood pressure is something you don`t notice you have. If you leave it untreated/undertreated for a long time, it can damage your internal organs, which can lead to serious consequences; examples are heart attack, heart failure, kidney failure and stroke. These diseases may affect your quality of life, and in worst case you may die from it.»

Finally, the doctor gives general advice:

**Advice, tips and prevention**
«Do you know how to take your medication? /Do you know how to use your drugs?

In addition to taking your medication, there are several measures you can do to reduce your blood pressure; including a healthy diet and exercise. Particularly a high intake of salt may worsen the blood pressure, and even lead to a suboptimal effect of some of your medications (diuretics). Do you have any questions regarding this?»

**Online Suppl. Text §5**

**Blood Pressure Measurements**

Blood pressure measurement devices used were: Watch BP 03, Microlife, Cambridge, UK (Oslo/Tromsø); Sun Tech Oscar 2, Sun Tech Medical, Morrisville, NC, USA (Trondheim); OMRON HEM-907, OMRON Healthcare, Kyoto, Japan (Bergen).

**Online Suppl. Text §6**

**Questionnaires**

Patient-reported adherence to medications was based on a five-item questionnaire where the patient answered “never”, “rarely”, “mostly” or “always” to each question. The first question: “I take my blood pressure medication as agreed with my doctor”, was used as an indicator of patient-reported adherence. The last three questions were taking non-intentional non-adherence into account, and a total sum based on all four items were calculated. The lowest possible score 5, indicated excellent adherence, 25 indicated complete non-adherence.

Patient-reported side effects of antihypertensive medications were registered on a list consisting of 26 potential side effects and graded on a severity scale ranging from 1-5; 1 being mild symptoms and 5 severe. A total sum of side effects was calculated by adding the individual scores of all 26 potential side effects, giving a minimum score of 0 and a maximum score of 130.

**Copy list of side effects**

**REGISTRATION OF SIDE EFFECTS**

On this form we ask you to register changes in your health that may be related to side effects of your antihypertensive medication. If possible, write down the specific drug you think is responsible for this side-effect.

Do you experience side effects of your current antihypertensive medication? Yes No

If yes, describe; ­­­­­­­­­­­­­­­­­­­­__________________________________________________________________________________

|  | **SIDE EFFECT** | **INTERMITTENT** | **PERSISTENT** | **SCALE**  1=mild 5=severe | **DRUG** |
| --- | --- | --- | --- | --- | --- |
|  | **Feeling tired/weak** |  |  | **1 2 3 4 5** |  |
|  | **Dizziness** |  |  | **1 2 3 4 5** |  |
|  | **Fainting** |  |  | **1 2 3 4 5** |  |
|  | **Headache** |  |  | **1 2 3 4 5** |  |
|  | **Muscle cramps** |  |  | **1 2 3 4 5** |  |
|  | **Concentration difficulties** |  |  | **1 2 3 4 5** |  |
|  | **Mood changes** |  |  | **1 2 3 4 5** |  |
|  | **Sleep disturbances** |  |  | **1 2 3 4 5** |  |
|  | **Cold hands and/or feet** |  |  | **1 2 3 4 5** |  |
|  | **Raynaud’s phenomenon** |  |  | **1 2 3 4 5** |  |
|  | **Palpitations** |  |  | **1 2 3 4 5** |  |
|  | **Chest pain** |  |  | **1 2 3 4 5** |  |
|  | **Dry cough** |  |  | **1 2 3 4 5** |  |
|  | **Difficulties breathing** |  |  | **1 2 3 4 5** |  |
|  | **Diarrhea/loose stools** |  |  | **1 2 3 4 5** |  |
|  | **Nausea/vomit** |  |  | **1 2 3 4 5** |  |
|  | **Constipation** |  |  | **1 2 3 4 5** |  |
|  | **Dry mouth** |  |  | **1 2 3 4 5** |  |
|  | **Weight change** |  |  | **1 2 3 4 5** |  |
|  | **Dry eyes** |  |  | **1 2 3 4 5** |  |
|  | **Skin ailments (rash and/or itching)** |  |  | **1 2 3 4 5** |  |
|  | **Sexual problems** |  |  | **1 2 3 4 5** |  |
|  | **Overhydration, swollen legs** |  |  | **1 2 3 4 5** |  |
|  | **Frequent urination** |  |  | **1 2 3 4 5** |  |
|  | **Yellow discoloration of skin/eyes** |  |  | **1 2 3 4 5** |  |
|  | **Other:** |  |  | **1 2 3 4 5** |  |

**Online Suppl. Text §7**

**Power calculations**

The rationale for anticipating 8 mmHg difference between the groups rather than 4 mmHg as in most drug trials with a single new drug typically added, was that the patients had to have ≥2 different drug classes prescribed to be included in the study, and a majority (67%) used single pill combinations. We therefore would expect the BP to decrease by at least 8 mmHg if they started to take their prescribed drugs. In nine studies comparing major two-drug combinations with placebo the difference in SBP varied from 23 to 5.6 mmHg, with a mean value of 8.6 mmHg (Table 21 in the ESC/ESH 2018 Hypertension Guidelines)^(1)^. The experience that more than 75% of patients require 2 or more drugs to achieve BP goal^(2)^ is the basis for the recommended use of early combination therapy in modern hypertension guidelines.^(1)^

Withdrawals were considered but sample size not adjusted for two main reasons; (i) with n=80 enrolled patients we expected a higher than strictly needed statistical power to show TDM benefit based on previous research ^(3, 4)^, and (ii) we have through 40 years of this type of mechanistic drug research not experienced substantial amount of withdrawals.

**Online Suppl. Text §8**

**Self-reported Adherence and Reactions to TDM**

All patients but one in the TDM group self-reported good adherence, and there was no significant difference between groups at inclusion, p=0.46 (Table 1). Self-reported adherence remained unchanged throughout the study with a median score of six (on a scale from five to 25, where five corresponded to excellent adherence) in both groups at screening, three months and six months, p=0.95.

After giving information about serum concentrations, the investigators noted the patients’ reactions to TDM for 23 of the 26 patients; 13 patients admitted sub-optimal adherence and 10 patients were uncomprehending or denied non-adherence. Most patients’ moods were indifferent after being informed (78%). However, (i) three patients were embarrassed, (ii) one patient showed symptoms of stress and (iii) one patient despaired because they experienced that capsules appeared in their stool undigested. One patient’s BP increased at three months (daytime systolic ABPM 146 to 189 mmHg), but the other four patients with an emotional response to TDM-information showed a significant decrease in systolic daytime ABPM at three months compared to those with indifferent mood; SBP reduction 21 (±4) vs. 8 (±9) mmHg (p=0.012).

Although the number of patients was limited, an emotional reaction to the TDM-information seemed to be associated with a greater reduction in BP, indicating individual differences in the response to TDM.

**Online Suppl. Text §9**

**Pharmacological considerations**

Our definition of non-adherence may potentially have misclassified some patients with sub-optimal adherence as adherent. We aimed to minimize patients’ instructions as much as possible before the first visit, not to interfere with their usual routine (or absence of routine) for medication intake. Most of our patients reported intake of medications two-six hours before measurements of serum concentrations, corresponding to a peak concentration level (Cmax) of most drugs. There are large fluctuations in concentrations around peak time leading to uncertainty whether one measures a rising, peak or falling concentration, leading to wide concentration ranges for samples taken at this time-point, also overlapping the trough (Cmin) concentration ranges. Based on this knowledge we chose a strict definition of adherence, namely the lower limit of the expected trough concentration range at steady state as cut-off for non-adherence. These concentrations are based on intake of the lowest recommended prescribed dose combined with the highest clearance for all but depot formulas. For depot formula drugs, the lower level of average concentrations was applied^(5)^. Thus, patients with suboptimal adherence, who had taken their drugs only once or twice before attending the study may have been misclassified as adherent. Some studies use an even stricter definition of adherence by using the lower limit of detection as a cut-off. We may have included some few patients with partial or non-intentional poor adherence (e.g. did not take their morning doses before the screening visit as a precaution due to the study and not due to non-adherence) that wouldn`t have been included by this definition.

White-coat adherence, i.e. intake of medication just before the visit, may accidentally have been encouraged in the intervention group during follow-up since these patients were informed about the possibility of serum measurements of antihypertensive drugs. This challenge supports the necessity of repeated concentration measurements or other methods to reveal non-adherence, e.g. directly observed therapy (DOT), to find patients who do not take their drugs regularly^(6)^.

TDM has been shown as a promising and cost-effective^(7)^ method for evaluating non-adherence in patients with uncontrolled hypertension^(8)^. Therapeutic reference ranges for optimal antihypertensive effect of drugs are not yet established, but in the future TDM may offer the possibility to individualize doses and antihypertensive treatment, e.g. by dried blood spot sampling at the doctor’s office or by the patient at home^(9)^.

A general limitation is that cut-off limits for defining non-adherence have not been well established. We chose to use the lower limits of the calculated trough (Cmin) concentration ranges, corresponding to what you would expect to measure in a patient 12-24 h after intake of a low dose of a medication, given a high metabolism of the drug. By using this definition, we may have included some patients that wouldn`t have been included by using another cut-off, like the limit of quantification as the definition of non-adherence.

**TABLES**

**Table S1. Overview of Antihypertensive Agents and Serum Cut-off Values^(5, 10)^**

| **Antihypertensive agents** | **Analytical calibration ranges^a^ (nmol/L)** | **Established**  **cut-off values**  **(nmol/L)** |
| --- | --- | --- |
| **Selective α-adrenoreceptor blockers** |  |  |
| Doxazosin^b^ | 10-500 | 10 |
| **Beta-blockers** |  |  |
| Atenolol | 20-6000 | 75 |
| Bisoprolol | 1-1000 | 10 |
| Carvedilol | 1-1000 | 5 |
| Labetolol | 1-1000 | 50 |
| Metoprolol^b^ | 10-3000 | 10 |
| **Calcium channel blockers** |  |  |
| Amlodipine | 1-1000 | 5 |
| Diltiazem^b^ | 10-1000 | 150 |
| Lercanidipine | 0.1-10 | 0.15 |
| Nifedipine^b^ | 1-1000 | 10 |
| Verapamil^b^ | 9-924 | 50 |
| **ACE inhibitors** |  |  |
| Enalaprilat^c^ | 1-1000 | 10 |
| Lisinopril | 10-500 | 10 |
| Ramiprilat^c^ | 1-1000 | 4 |
| **Angiotensin II receptor blockers** |  |  |
| Candesartan | 1-1000 | 15 |
| Irbesartan | 240-20000 | 400 |
| Losartan carboxylic acid^c^ | 10-3000 | 30 |
| Telmisartan | 4-6000 | 10 |
| Valsartan | 240-20000 | 100 |
| **Thiazide diuretics** |  |  |
| Bendroflumethiazide | 10-500 | 2.5 |
| Hydrochlorothiazide | 10-3000 | 10 |
| **Aldosterone antagonists** |  |  |
| Canrenone^c^ | 1-1000 | 30 |
| Eplerenone | 20-6000 | 3.5 |

ACE=Angiotensin Converting Enzyme

^a^For each drug six calibrators, quadratic or linear models of fit for the calibration curves were applied.

^b^Depot drugs, calculated using average concentration.

^c^Metabolites of the antihypertensive agents (enalaprilat; enalapril, ramiprilat; ramipril, losartan carboxylic acid; losartan, canrenone; spironolactone)

**Table S2. Adherence and blood pressures at three months and change from screening, including 4 patients lost to follow-up, using their last recorded BP`s and adherence evaluation.**

| Variable | TDM  group  (n=26) | Control  group  (n=20) | p-value |
| --- | --- | --- | --- |
| Adherent n (%) | 19 (73) | 10 (50) | 0.13 |
| Ambulatory daytime SBP, mmHg | 141.1 (±14.1) | 141.2 (±16.2) | 0.99 |
| Δ, mmHg | -6.7 (±14.5) | -5.9 (±12.1) | 0.83 |
| Ambulatory daytime DBP, mmHg | 86.7 (±10.9) | 82.8 (±12.8) | 0.28 |
| Δ, mmHg | -2.9 (±8.9) | -3.9 (±7.2) | 0.68 |
| Ambulatory daytime HR, bpm | 75.0 (±12.2) | 72.0 (±9.7) | 0.37 |
| Δ, bpm | -1.7 (±6.5) | -2.4 (±4.7) | 0.68 |
| Ambulatory 24-h SBP, mmHg | 136.8 (±12.4) | 138.2 (±15.4) | 0.74 |
| Δ, mmHg | -6.0 (±14.9) | -5.1 (±11.9) | 0.82 |
| Ambulatory 24-h DBP, mmHg | 82.4 (±9.5) | 80.2 (±11.0) | 0.46 |
| Δ, mmHg | -3.2 (±8.7) | -3.4 (±6.7) | 0.92 |
| Ambulatory 24-h HR, bpm | 72.4 (±11.5) | 71.1 (±9.1) | 0.67 |
| Δ, bpm | -1.1 (±5.4) | -1.3 (±5.1) | 0.91 |
| Ambulatory nighttime SBP, mmHg | 125.1 (±13.1) | 126.5 (±15.0) | 0.75 |
| Δ, mmHg | -2.9 (±18.9) | -5.5 (±13.3) | 0.61 |
| Ambulatory nighttime DBP, mmHg | 72.3 (±8.5) | 71.4 (±9.1) | 0.69 |
| Δ, mmHg | -2.1 (±10.3) | -3.4 (±7.8) | 0.66 |
| Ambulatory nighttime HR, bpm | 65.6 (±11.1) | 67.3 (±10.9) | 0.60 |
| Δ,bpm | 0.7 (±4.8) | -0.1 (±7.0) | 0.69 |
| Office SBP, mmHg | 143.4 (±16.1) | 143.0 (±20.8) | 0.94 |
| Δ, mmHg | -13.3 (±20.2) | -11.4 (±17.5) | 0.73 |
| Office DBP, mmHg | 85.2 (±13.3) | 85.8 (±13.7) | 0.90 |
| Δ, mmHg | -9.0 (±14.2) | -8.5 (±9.9) | 0.89 |
| Office HR, bpm | 72.7 (±12.7) | 72.8 (±12.0) | 0.99 |
| Δ,bpm | 1.9 (±7.8) | 0.9 (±6.3) | 0.63 |
| Ambulatory daytime SBP <135, n (%) | 6 (23.1) | 8 (40.0) | 0.36 |

Results are reported as n (%) or mean (±SD), p-value denotes difference between the groups. Abbreviations: Δ, change; SBP, systolic blood pressure; DBP, diastolic blood pressure; HR, heart rate.

**Table S3. Adherence and blood pressures at six months and change from screening.**

| Variable | TDM  group (n=23) | Control group  (n=12)^a^ | p-value |
| --- | --- | --- | --- |
| Adherent, n (%) | 20 (87) | 8 (62) | 0.11 |
| Ambulatory daytime SBP, mmHg | 136.4 (±15.7) | 135.0 (±14.2) | 0.80 |
| Δ, mmHg | -10.5 (±16.6) | -9.3 (±13.0) | 0.82 |
| Ambulatory daytime DBP, mmHg | 83.1 (±10.0) | 81.8 (±9.3) | 0.70 |
| Δ, mmHg | -7.0 (±10.3) | -5.3 (±8.7) | 0.64 |
| Ambulatory daytime HR, bpm | 73.2 (±11.9) | 72.1 (±9.4) | 0.78 |
| Δ, bpm | -4.2 (±6.5) | -2.1 (±5.9) | 0.36 |
| Ambulatory 24-h SBP, mmHg | 132.5 (±14.8) | 130.8 (±13.6) | 0.74 |
| Δ, mmHg | -9.4 (±15.6) | -8.7 (±12.4) | 0.89 |
| Ambulatory 24-h DBP, mmHg | 79.8 (±9.3) | 78.1 (±9.2) | 0.62 |
| Δ, mmHg | -6.4 (±9.3) | -5.3 (±8.8) | 0.76 |
| Ambulatory 24-h HR, bpm | 71.1 (±11.7) | 70.1 (±8.9) | 0.81 |
| Δ, bpm | -3.5 (±4.9) | -1.3 (±5.2) | 0.25 |
| Ambulatory nighttime SBP, mmHg | 122.5 (±15.8) | 119.8 (±14.6) | 0.62 |
| Δ, mmHg | -5.0 (±16.8) | -8.0 (±12.9) | 0.60 |
| Ambulatory nighttime DBP, mmHg | 70.6 (±9.4) | 68.3 (±9.4) | 0.51 |
| Δ, mmHg | -4.3 (±10.3) | -5.6 (±10.1) | 0.73 |
| Ambulatory nighttime HR, bpm | 64.6 (±11.0) | 65.2 (±7.7) | 0.87 |
| Δ, bpm | -1.3 (±3.8) | 0.7 (±4.7) | 0.19 |
| Office SBP, mmHg | 138.4 (±14.5) | 136.0 (±13.9) | 0.63 |
| Δ, mmHg | -18.3 (±25.1) | -17.7 (±13.0) | 0.93 |
| Office DBP, mmHg | 84.5 (±10.4) | 84.9 (±11.2) | 0.93 |
| Δ, mmHg | -10.5 (±13.0) | -8.9 (±5.8) | 0.63 |
| Office HR, bpm | 70.1 (±13.3) | 76.0 (±12.1) | 0.20 |
| Δ, bpm | -2.2(±7.6) | 4.4 (±7.4) | 0.02 |
| Ambulatory daytime SBP <135, n (%) | 10 (44) | 7 (58) | 0.49 |

Results are reported as n (%) or mean (±SD), p-value denotes difference between the groups. ^a^Office BP and adherence evaluation performed in 13 patients, ABPM missing for one patient in the control group at the 6-month visit. Abbreviations: Δ, change; SBP, systolic blood pressure; DBP, diastolic blood pressure; HR, heart rate.

**Table S4. Adherence and blood pressures at six months and change from screening, including 11 patients lost to follow-up, using their last recorded BP`s and adherence evaluation.**

| Variable | TDM  group (n=26) | Control group  (n=20) | p-value |
| --- | --- | --- | --- |
| Adherent, n (%) | 21 (81) | 10 (50) | 0.06 |
| Ambulatory daytime SBP, mmHg | 139.0 (±18.1) | 141.4 (±17.3) | 0.66 |
| Δ, mmHg | -8.8 (±19.0) | -5.7 (±13.7) | 0.53 |
| Ambulatory daytime DBP, mmHg | 83.5 (±10.4) | 82.8 (±11.2) | 0.82 |
| Δ, mmHg | -6.0 (±11.5) | -4.0 (±8.0) | 0.49 |
| Ambulatory daytime HR, bpm | 73.2 (±11.2) | 73.4 (±8.3) | 0.95 |
| Δ, bpm | -3.5 (±6.6) | -1.0 (±5.5) | 0.18 |
| Ambulatory 24-h SBP, mmHg | 131.3 (±25.7) | 137.8 (±16.9) | 0.33 |
| Δ, mmHg | -11.4 (±27.3) | -5.4 (±13.1) | 0.37 |
| Ambulatory 24-h DBP, mmHg | 80.3 (±9.2) | 79.6 (±10.7) | 0.81 |
| Δ, mmHg | -5.2 (±10.1) | -4.0 (±7.8) | 0.64 |
| Ambulatory 24-h HR, bpm | 70.7 (±10.8) | 71.9 (±8.2) | 0.68 |
| Δ, mmHg | -2.8 (±4.9) | -0.5 (±4.) | 0.11 |
| Ambulatory nighttime SBP, mmHg | 125.3 (±16.6) | 126.2 (±16.3) | 0.85 |
| Δ, mmHg | -2.8 (±19.3) | -5.7 (±12.4) | 0.56 |
| Ambulatory nighttime DBP, mmHg | 71.4 (±9.0) | 70.7 (±9.7) | 0.81 |
| Δ, mmHg | -3.0 (±10.7) | -4.0 (±8.4) | 0.76 |
| Ambulatory nighttime HR, bpm | 63.4 (±10.4) | 68.4 (±10.2) | 0.15 |
| Δ, mmHg | -1.0 (±3.7) | 1.0 (±4.3) | 0.09 |
| Office SBP, mmHg | 141.2 (±17.6) | 142.1 (±18.4) | 0.86 |
| Δ, mmHg | -15.5 (±24.9) | -12.2 (±15.2) | 0.58 |
| Office DBP, mmHg | 84.6 (±11.3) | 86.3 (±13.3) | 0.65 |
| Δ, mmHg | -9.7 (±12.5) | -8.0 (±9.5) | 0.62 |
| Office HR, bpm | 70.0 (±12.5) | 75.7 (±12.1) | 0.12 |
| Δ, bpm | -0.8 (±8.3) | 3.8 (±6.9) | 0.05 |
| Ambulatory daytime SBP <135, n (%) | 10 (39) | 8 (40) | 1.00 |

Results are reported as n (%) or mean (±SD), p-value denotes difference between the groups. Abbreviations: Δ, change; SBP, systolic blood pressure; DBP, diastolic blood pressure; HR, heart rate.

**Table S5. Side effects registered during six months of follow up.**

| Variable | TDM  group | Control  group | p-value |
| --- | --- | --- | --- |
| Screening visit, n | 26 | 20 |  |
| Number of patients with side effect, n (%) | 16 (62) | 16 (80) | 0.21 |
| Number of side effects | 1.5 (0,4.75) | 2 (1.0,3.0) | 0.68 |
| Severity score | 3.5 (0,12.0) | 4 (1.0, 9.0) | 0.80 |
| 3-month visit, n | 18 | 14 |  |
| Number of patients with side effect, n (%) | 18 (70) | 14 (82) | 0.49 |
| Number of side effects | 2 (0,4.0) | 2 (1.0,5.0) | 0.49 |
| Severity score | 3 (0,9.5) | 6 (1.5,15.0) | 0.21 |
| 6-month visit, n | 16 | 11 |  |
| Number of patients with side effect, n (%) | 16 (70) | 11 (85) | 0.44 |
| Number of side effects | 2 (0,5.0) | 2 (1.0, 5.5) | 0.47 |
| Severity score | 4 (0,10.0) | 6 (1.5,17.0) | 0.21 |

Results are reported as n (%) or median (IQR), p-value denotes difference between the intervention and the control group. There was no change in the severity score between groups over time (p=0.96).

**FIGURES**

**Figure S1.** Office BP in the TDM group (red) and control group (blue) during six months follow-up.


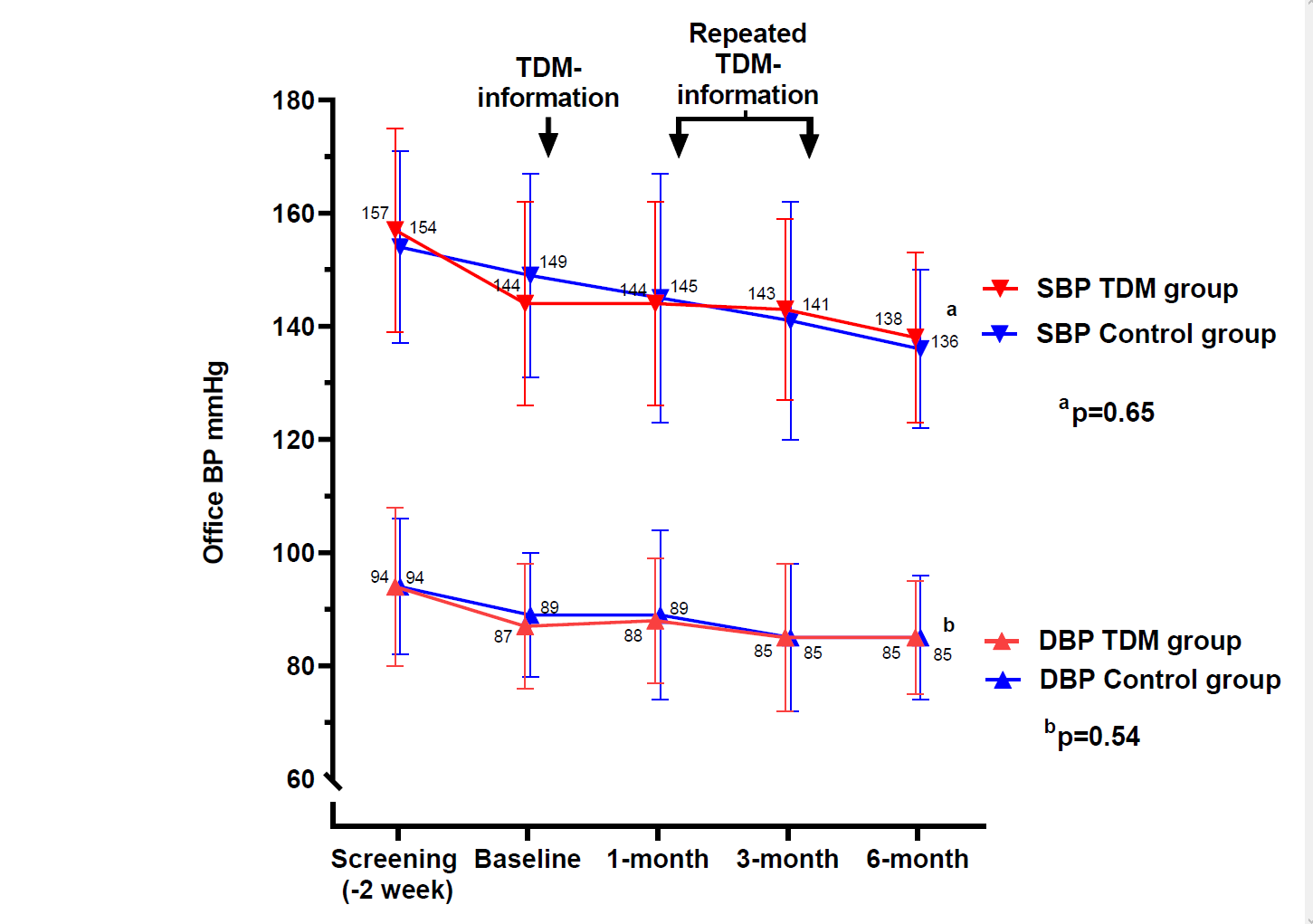


B

A


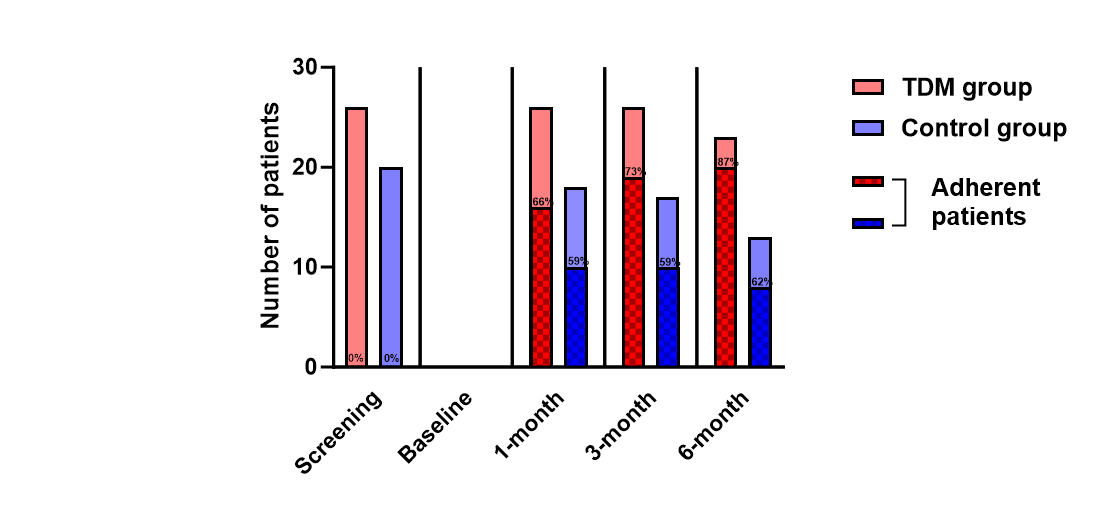


Panel A: Visits are marked on the X-axis and mean blood pressure (mmHg) is shown on the Y-axis. The Y-axis is truncated. The mixed model analysis of variance (ANOVA) showed that daytime systolic ABPM (p=0.65)^a^ and diastolic ABPM (p=0.54)^b^ did not differ between the groups.

Panel B: Number and fractions of adherent patients at each visit.

Abbreviations: ABPM=ambulatory blood pressure measurement; SBP=systolic blood pressure; DBP=diastolic blood pressure; TDM=therapeutic drug monitoring.

**Figure S2.** Individual data for Daytime ABPM (panel A), Nighttime ABPM (panel B), 24-h ABPM (panel C) and office BP (panel D) during six months follow-up.
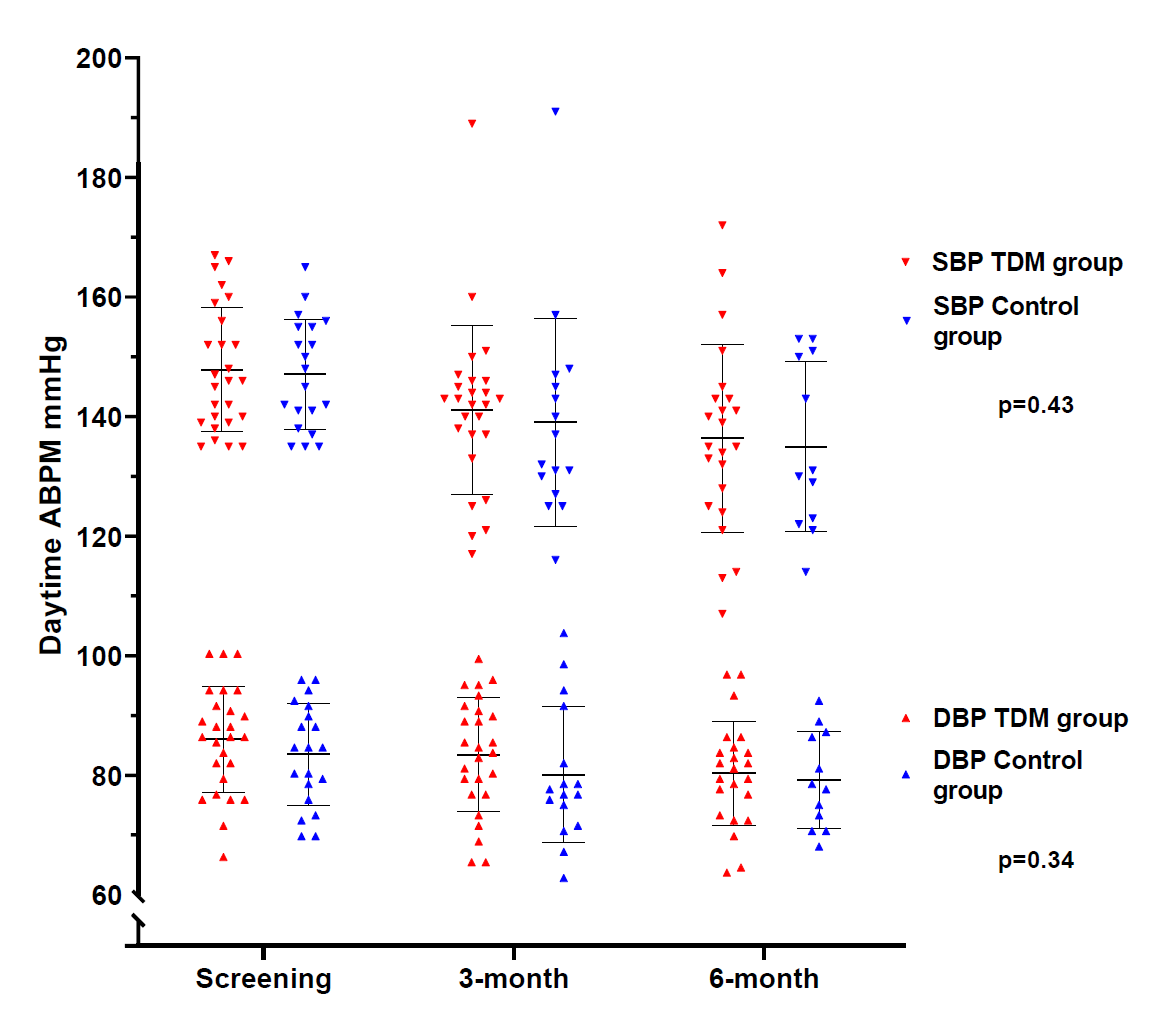

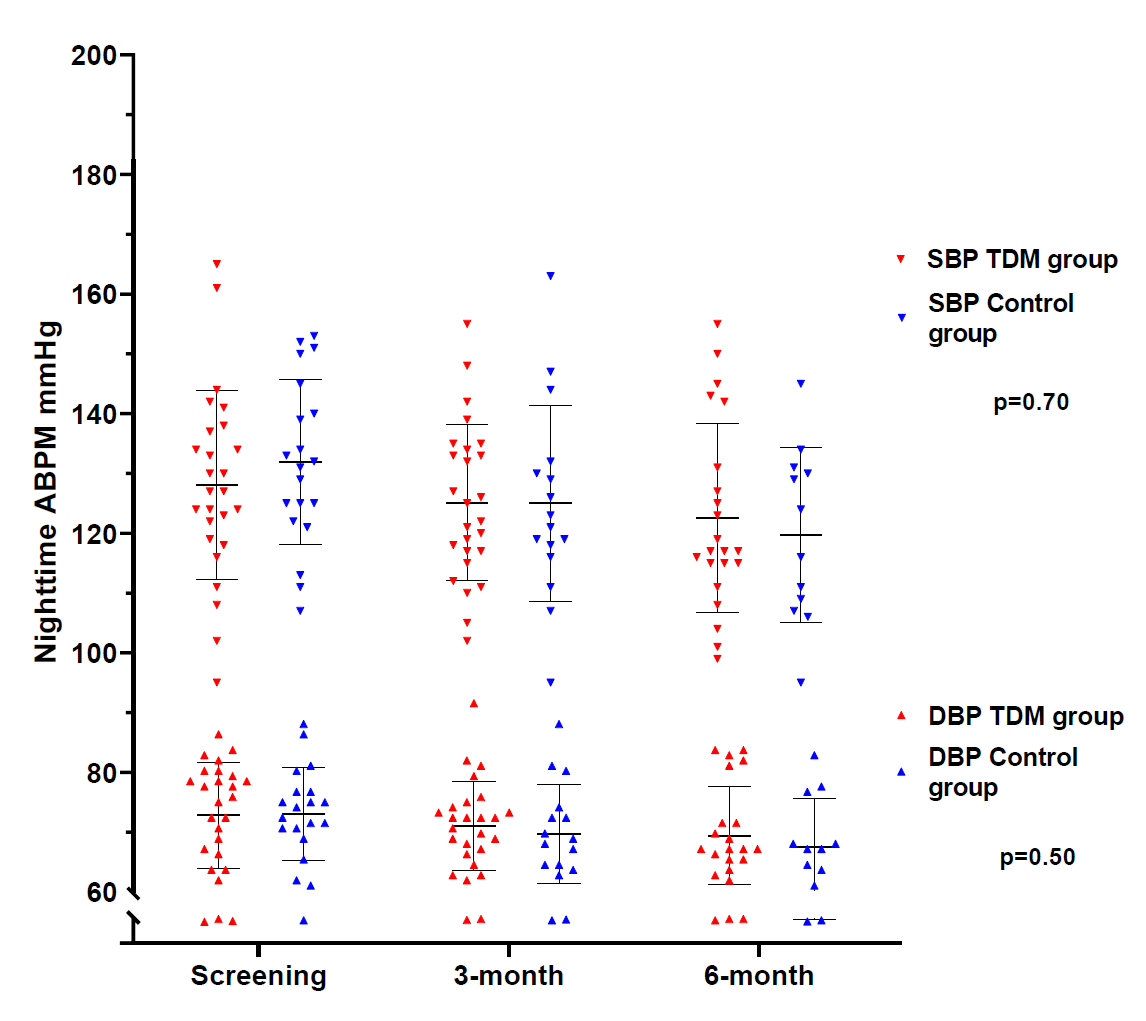


B

A


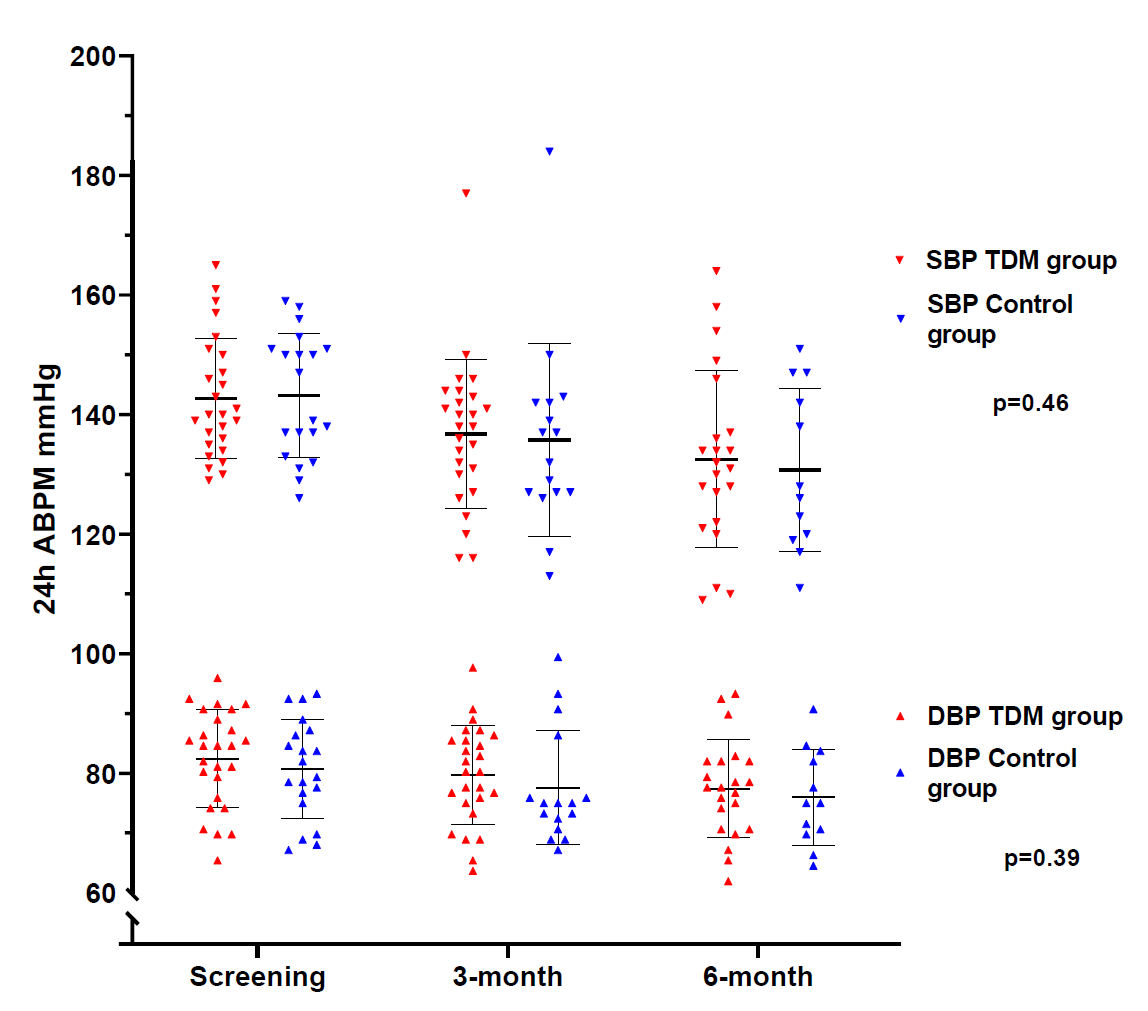

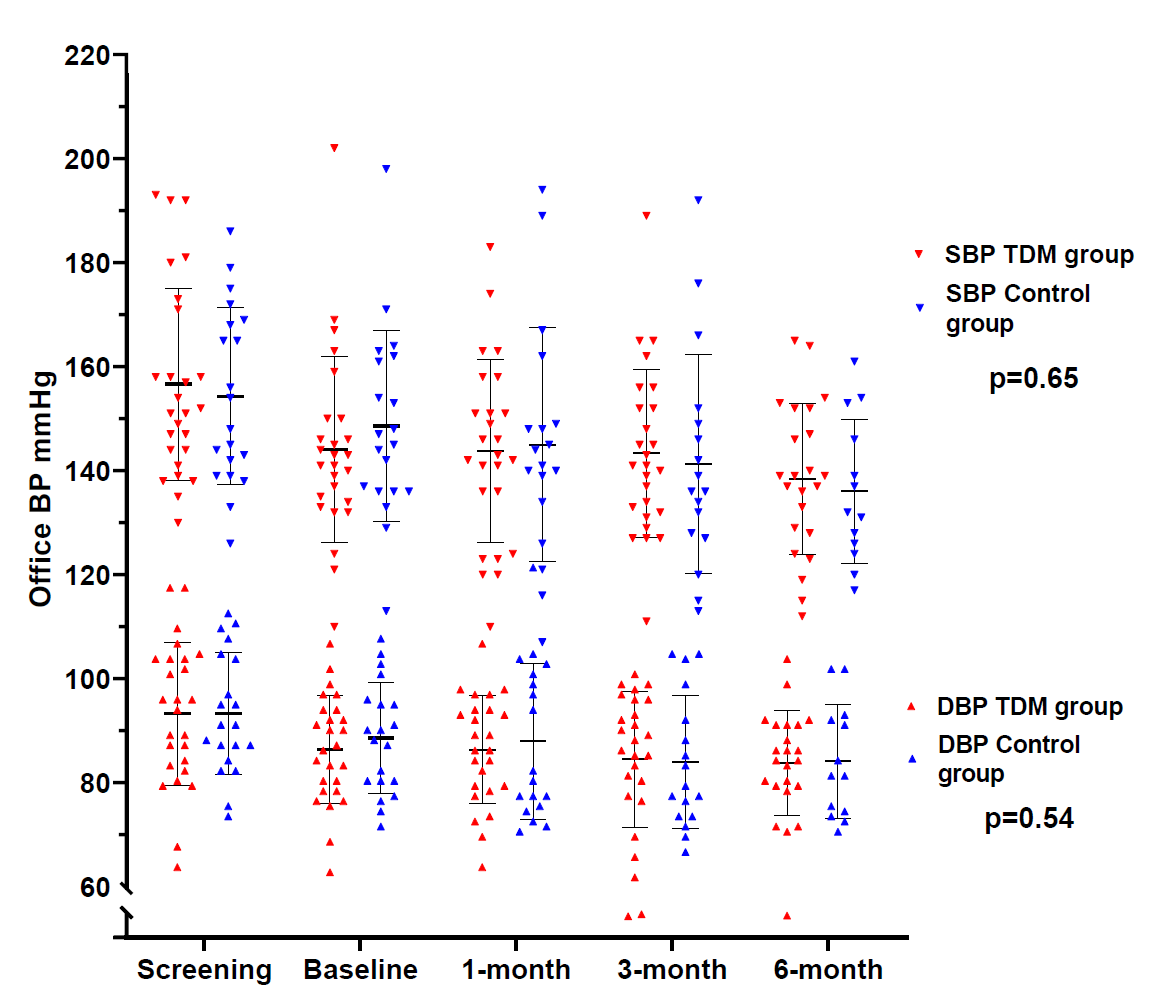


C

D

The intervention group is marked in red and the control group in blue. Visits are marked on the X-axis and blood pressure (mmHg) is shown on the Y-axis. The Y-axis is truncated. Mean (±SD) is marked for each group at each visit. The mixed model analysis of variance (ANOVA) showed that BPs did not differ between the groups.

Abbreviations: ABPM=ambulatory blood pressure measurement; SBP=systolic blood pressure; DBP=diastolic blood pressure; TDM=therapeutic drug monitoring

**Figure S3.** Individual data for Systolic Daytime ABPM and Adherence status


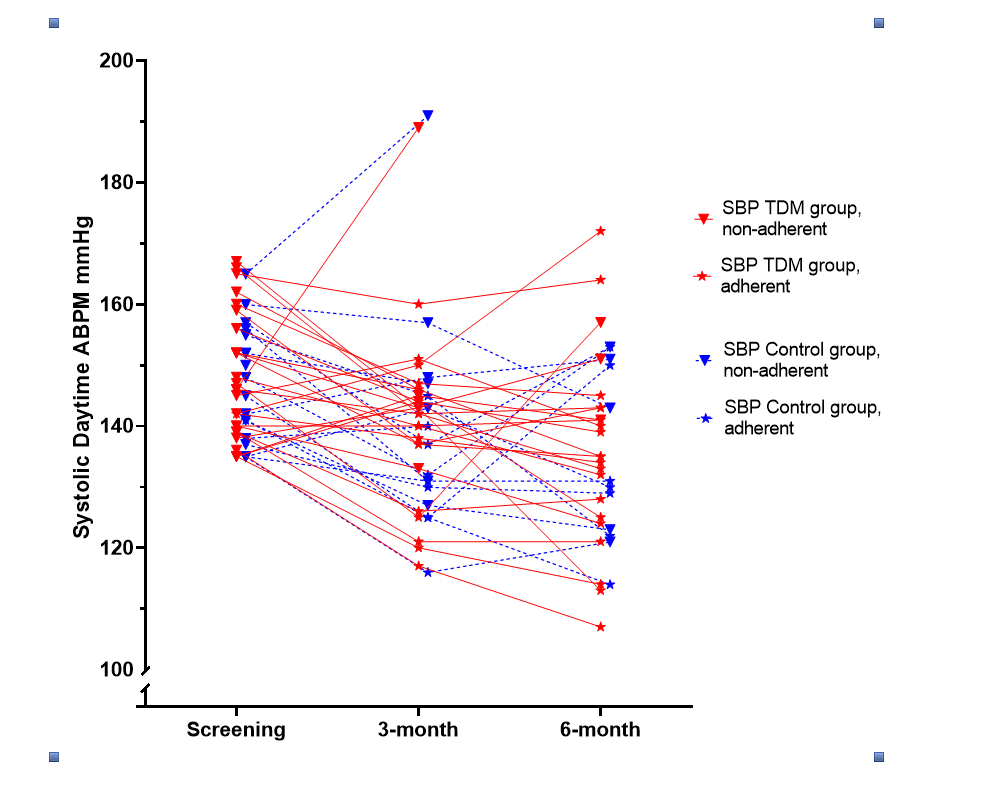


Changes in systolic daytime ABPM and adherence status in the intervention (red) and control (blue) groups. Visits are marked on the X-axis, and SBP on the Y-axis (mmHg). The Y-axis is truncated. A star indicates that the patient is evaluated as adherent.

*Abbreviations*: ABPM=ambulatory blood pressure measurement; SBP=systolic blood pressure

**References**

1. Williams B, Mancia G, Spiering W, Agabiti Rosei E, Azizi M, Burnier M, Clement DL, Coca A, de Simone G, Dominiczak A, Kahan T, Mahfoud F, Redon J, Ruilope L, Zanchetti A, Kerins M, Kjeldsen SE, Kreutz R, Laurent S, Lip GYH, McManus R, Narkiewicz K, Ruschitzka F, Schmieder RE, Shlyakhto E, Tsioufis C, Aboyans V, Desormais I. 2018 ESC/ESH Guidelines for the management of arterial hypertension: The Task Force for the management of arterial hypertension of the European Society of Cardiology and the European Society of Hypertension: The Task Force for the management of arterial hypertension of the European Society of Cardiology and the European Society of Hypertension. J Hypertens. 2018;36(10):1953-2041.

2. Mensah GA, Bakris G. Treatment and Control of High Blood Pressure in Adults. Cardiology Clinics. 2010;28(4):609-22.

3. Brinker S, Pandey A, Ayers C, Price A, Raheja P, Arbique D, Das SR, Halm EA, Kaplan NM, Vongpatanasin W. Therapeutic drug monitoring facilitates blood pressure control in resistant hypertension. Journal of the American College of Cardiology. 2014;63(8):834-5.

4. Gupta P, Patel P, Strauch B, Lai FY, Akbarov A, Gulsin GS, Beech A, Maresova V, Topham PS, Stanley A, Thurston H, Smith PR, Horne R, Widimsky J, Keavney B, Heagerty A, Samani NJ, Williams B, Tomaszewski M. Biochemical Screening for Nonadherence Is Associated With Blood Pressure Reduction and Improvement in Adherence. Hypertension. 2017;70(5):1042-8.

5. Rognstad S, Søraas CL, Bergland OU, Høieggen A, Strømmen M, Helland A, Opdal MS. Establishing Serum Reference Ranges for Antihypertensive Drugs. Ther Drug Monit. 2021;43(1):116-25.

6. Halvorsen LV, Bergland OU, Søraas CL, Larstorp ACK, Hjørnholm U, Kjær VN, Kringen MK, Clasen PE, Haldsrud R, Kjeldsen SE, Rostrup M, Elmula FEMF, Opdal MS, Høieggen A. Nonadherence by Serum Drug Analyses in Resistant Hypertension: 7‐Year Follow‐Up of Patients Considered Adherent by Directly Observed Therapy. J Am Heart Assoc. 2022;11(18):e025879.

7. Chung O, Vongpatanasin W, Bonaventura K, Lotan Y, Sohns C, Haverkamp W, Dorenkamp M. Potential cost-effectiveness of therapeutic drug monitoring in patients with resistant hypertension. J Hypertens. 2014;32(12):2411-21.

8. Ceral J, Habrdova V, Vorisek V, Bima M, Pelouch R, Solar M. Difficult-to-control arterial hypertension or uncooperative patients? The assessment of serum antihypertensive drug levels to differentiate non-responsiveness from non-adherence to recommended therapy. Hypertens Res. 2011;34(1):87-90.

9. Peeters LEJ, Bahmany S, Dekker T, Aliawi A, van Domburg B, Versmissen J, Koch BCP. Development and Validation of a Dried Blood Spot Assay Using UHPLC-MS/MS to Identify and Quantify 12 Antihypertensive Drugs and 4 Active Metabolites: Clinical Needs and Analytical Limitations. Ther Drug Monit. 2022;44(4):568-77.

10. Bergland OU, Halvorsen LV, Søraas CL, Hjørnholm U, Kjær VN, Rognstad S, Brobak KM, Aune A, Olsen E, Fauchald YM, Heimark S, Thorstensen CW, Liestøl K, Solbu MD, Gerdts E, Mo R, Rostrup M, Kjeldsen SE, Høieggen A, Opdal MS, Larstorp ACK, Fadl Elmula FEM. Detection of Nonadherence to Antihypertensive Treatment by Measurements of Serum Drug Concentrations. Hypertension. 2021;78(3):617-28.
